# Insight into Dominant Cellulolytic Bacteria from Two Biogas Digesters and Their Glycoside Hydrolase Genes

**DOI:** 10.1371/journal.pone.0129921

**Published:** 2015-06-12

**Authors:** Yongjun Wei, Haokui Zhou, Jun Zhang, Lei Zhang, Alei Geng, Fanghua Liu, Guoping Zhao, Shengyue Wang, Zhihua Zhou, Xing Yan

**Affiliations:** 1 CAS-Key Laboratory of Synthetic Biology, Institute of Plant Physiology and Ecology, Shanghai Institutes for Biological Sciences, Chinese Academy of Sciences, Shanghai, China; 2 Department of Microbiology, the Chinese University of Hong Kong, the Prince of Wales Hospital, Hong Kong, China; 3 Shanghai-MOST Key Laboratory for Health and Disease Genomics, Chinese National Human Genome Center, Shanghai, China; Free University of Bozen/Bolzano, ITALY

## Abstract

Diverse cellulolytic bacteria are essential for maintaining high lignocellulose degradation ability in biogas digesters. However, little was known about functional genes and gene clusters of dominant cellulolytic bacteria in biogas digesters. This is the foundation to understand lignocellulose degradation mechanisms of biogas digesters and apply these gene resource for optimizing biofuel production. A combination of metagenomic and 16S rRNA gene clone library methods was used to investigate the dominant cellulolytic bacteria and their glycoside hydrolase (GH) genes in two biogas digesters. The 16S rRNA gene analysis revealed that the dominant cellulolytic bacteria were strains closely related to *Clostridium straminisolvens* and an uncultured cellulolytic bacterium designated BG-1. To recover GH genes from cellulolytic bacteria in general, and BG-1 in particular, a refined assembly approach developed in this study was used to assemble GH genes from metagenomic reads; 163 GH-containing contigs ≥ 1 kb in length were obtained. Six recovered GH5 genes that were expressed in *E*. *coli* demonstrated multiple lignocellulase activities and one had high mannanase activity (1255 U/mg). Eleven fosmid clones harboring the recovered GH-containing contigs were sequenced and assembled into 10 fosmid contigs. The composition of GH genes in the 163 assembled metagenomic contigs and 10 fosmid contigs indicated that diverse GHs and lignocellulose degradation mechanisms were present in the biogas digesters. In particular, a small portion of BG-1 genome information was recovered by PhyloPythiaS analysis. The lignocellulase gene clusters in BG-1 suggested that it might use a possible novel lignocellulose degradation mechanism to efficiently degrade lignocellulose. Dominant cellulolytic bacteria of biogas digester possess diverse GH genes, not only in sequences but also in their functions, which may be applied for production of biofuel in the future.

## Introduction

Biogas digesters can efficiently degrade lignocellulose and have long been used in the treatment of animal manure and agriculture residues[1]. Biogas production from lignocellulose is a complex process that includes four main steps: hydrolysis, acidogenesis, acetogenesis, and methanogenesis [2]. Previous studies have revealed that the hydrolysis step, in which lignocellulose is decomposed into soluble sugar by cellulolytic bacteria, is the rate-limiting step in biogas production [3]. In previous studies, the cellulolytic bacteria *Clostridium thermocellum*, *Clostridium stercorarium*, *Clostridium cellulolyticum*, and *Bacteroides cellulosolvens* were detected in various biogas digesters by culture-independent methods (i.e., 16S rRNA gene-based methods), suggesting that these microorganisms could be the dominant cellulolytic bacteria in many biogas digesters [4–9]. In addition to these microorganisms, uncultured cellulolytic bacteria from *Clostridia* group 4 were identified as dominant cellulolytic bacteria in a landfill-derived biogas digester through DNA-stable isotope probing (DNA-SIP) and fluorescence *in situ* hybridization (FISH) techniques [10]. Recently, a meta-analysis of microbial diversity in anaerobic digesters further confirmed that species of the genera *Acetivibrio* and *Clostridium* are the most abundant cellulolytic bacteria in anaerobic digesters [11]. However, due to the absence of corresponding isolates, the glycoside hydrolase (GH) genes and lignocellulose degradation mechanisms of several dominant cellulolytic bacteria are completely unknown.

In previous investigations in which metagenomic methods were used to characterize the microbial communities of biogas digesters, many metagenomic reads or assembled metagenomic contigs were assigned to *C*. *thermocellum*, demonstrating that bacteria similar to *C*. *thermocellum* were the dominant cellulolytic bacteria in these biogas digesters [12–14]. For example, in one metagenomic study of a production-scale biogas plant fermenter, more than 3.37% of the assembled contigs were binned to the genome of *C*. *thermocellum* [12]. However, due to the complex microbial composition and low coverage of the obtained metagenomic reads, as well as the low homology of the GH genes [15], full-length GH genes were rarely recovered from the short metagenomic reads [12]. Moreover, it was impossible to recover genome information from uncultured cellulolytic bacteria in the biogas digesters, because most of the short metagenomic reads and assembled contigs could not be assigned to known species [12].

In parallel, functional screenings of lignocellulase genes from metagenomic libraries of biogas digesters have also been performed in the past few years [16–19]. In one study, as many as 973 positive fosmid clones harboring lignocellulase genes were screened out from the metagenomic library of biogas digester Z7 [19]. Based on these results, lignocellulase genes or gene clusters that have potential uses in industry may be obtained through functional screening methods [16]. However, as seen in other studies [20, 21], some lignocellulase genes, including those from dominant cellulolytic bacteria, may be overlooked by functional screening, while other rare lignocellulase genes may be recovered, because positive clone selection is based on whether the GH genes are expressed in a host system (usually *Escherichia coli*). Even if all GH genes from all cellulolytic bacteria are recovered from the fosmid clones, there still remains the problem of linking these GH genes or gene clusters to the cellulolytic bacteria from which they were derived, as many GH genes are novel and are often assigned to uncultured bacteria [22]. Recently, methods such as PhyloPythiaS analysis, have been used to analyze diverse metagenomic samples through assigning metagenomic sequences from novel genera or higher-level clades to the proper taxonomy with limited reference data, [23–25].

In this study, 16S rRNA gene-based clone library methods and metagenomic methods were used to obtain insight into the dominant cellulolytic bacteria and their associated GH genes from two biogas digesters fed with pig manure and rice straw. A refined assembly approach developed in this study was utilized to assemble the dominant GH genes obtained from the biogas digesters. Using a sequence-based method, fosmid clones harboring either GH genes or the 16S rRNA gene of a dominant cellulolytic bacterium were obtained and sequenced. Using these methods, contigs from dominant cellulolytic bacteria were recovered, and information regarding the genome of the cellulolytic bacteria in the biogas digesters was revealed. Additionally, six recovered GH5 genes were expressed in *E*. *coli* and confirmed to have diverse lignocellulase activities.

## Materials and Methods

### Setup of two laboratory-scale biogas digesters and metagenomic DNA preparation

Two laboratory-scale 2.5-L biogas digesters (Z7 and Z8) were established with same slurry from a biogas plant fed with pig manure and operated at mesophilic temperature, located in Qianwei Village, Chongming County, Shanghai, China [7]. The two digesters were operated anaerobically with the same fed-batch fermentation method at 40°C. Every 7 days, 600 mL of biogas slurry were discharged from each biogas digester; after the discharge, 120 g of pig manure and 300 mL of slurry from the biogas plant, brought to a final volume of 600 mL with water, were fed into each biogas digester. An additional 3 g of rice straw were fed only to digester Z7 [16]. After the two biogas digesters had been running for 1.5 years, the microbial communities were stable as monitored by 16S rRNA gene PCR-DGGE (data not shown). The slurry samples were collected separately and subjected to DNA extraction as described previously [16]. The carbon and nitrogen content of the slurry and methane yield of the two biogas digesters were also measured (S1 File).

### Metagenomic sequencing, sequence analysis, and function annotation

DNA samples extracted from Z7 and Z8 were subjected to 454 pyrosequencing using the GS FLX sequencing system. Annotation of protein-coding reads was performed with BLASTX [26] against the NCBI-NR (http://www.ncbi.nlm.nih.gov/protein), KEGG [27], and STRING-COG [28] protein databases. A cutoff e-value of 10^–2^ was used to assign environmental gene tags (EGTs) [29] from the BLASTX results [30]. Functional profiles of the KEGG and COG functional categories were calculated from the number of EGTs assigned to the specific KO or COG. 16S rRNA genes were predicted from the metagenomic data using BLASTN against the RDPII database [31]. For each 16S rRNA gene match, reads with e-values ≤ 10^–5^, match lengths ≥ 65 nucleotides, and similarities ≥ 80% identity were retrieved for further analysis [30]. Reads encoding GH, carbohydrate-binding modules (CBM), and cellulosomal genes were determined according to their best hits from the CAZy [32] and NCBI databases (S1 File). MEGAN [33] was used to analyze the NCBI taxonomies of the metagenomic reads (S1 File). The 454 pyrosequencing reads from the two samples were deposited in the GenBank SRA database under GenomeProject ID #50503. Additionally, the Newbler software version 2.6 (Roche/454 Life Sciences) was used with default parameters (Minimum overlap length of 40 bp and Minimum overlap identity of 90%) to assemble the reads, and the assembled contigs ≥ 1 kb were deposited in NCBI database under accession numbers KJ797191- KJ801155.

### 16S rRNA gene-based analysis of microbial composition in the two biogas digesters

The microbial composition was analyzed by both PCR-based 16S rRNA gene clone library data and 454 pyrosequencing data (S1 File). The 16S rRNA gene sequence data was deposited in the GenBank database under accession numbers HQ155349–HQ156212 (Z7 sample) and HQ154667–HQ155348 (Z8 sample).

To compare phylogenetic profiles between the PCR amplicons and the metagenomic predictions in Z7 or Z8, sequences from both datasets were aligned with NAST at Greengenes [34], imported into the ARB software [35], and then inserted into the reference tree of the Greengenes database with the lanemaskPH filter using the parsimony insertion algorithm. From the resulting phylogenetic tree, groups of phyla were identified and the abundances of included sequences were calculated for the PCR amplicons and the metagenome, respectively.

### Recovery of GH family genes from two biogas digesters by a refined assembly approach

A refined assembly approach was used to recover dominant lignocellulase genes from short metagenomic reads obtained from the Z7 and Z8 samples using protein sequences from several GH families (GH1, GH3, GH5, GH9, GH10, and GH11) retrieved from the CAZy database as the reference database (S1 Fig) [32]. All of the 454 reads were queried against the reference database with BLASTX (e-value of 1e-2 and bacterial codon table-Q 11). The resulting high-scoring segment pair (HSP) hits were then subjected to assembly using the Geneious Pro Assembler (Biomatters, Auckland, New Zealand). To recover more genome information from neighboring genes, we also use the assembled GH-containing contigs to mega-BLAST the metagenomic reads and/or the contigs assembled from the total metagenomic reads by Newbler software (S1 Fig). Only contigs ≥ 1 kb in length were taken into account for further analysis. To predict the open reading frames (ORFs) and determine functional roles of the genes, FGENESB (http://linux1.softberry.com/berry.phtml) and BLASTX were used in combination to annotate the contigs. All contigs ≥ 1 kb in length were blasted (BLASTX) against known GH family protein sequences from the CAZy and Pfam databases. Further manual inspection was conducted to verify the assembly quality and annotation results to obtain the final list of recovered GH-containing contigs.

To test the accuracy of the assembled GH-containing contigs, 50 pairs of contig-specific primers were designed from 50 randomly selected assembled GH-containing contigs and used to amplify the corresponding PCR fragments from the same DNA sample used to generate the metagenomic data (S1 File; S1 Table). The 50 resultant PCR fragments were then sequenced and compared to sequences in the corresponding 50 assembled GH-containing contigs. The GenBank accession numbers of the recovered GH-containing contigs are KJ797028—KJ797190.

### Screening of positive fosmid clones harboring GH-containing contigs from the Z7 fosmid library

Contig-specific primers were also used to screen positive fosmid clones from the Z7 fosmid library (S1 Table). To obtain more information regarding the genome of the dominant bacterium BG-1 through PhyloPythiaS analysis [24], two additional fosmid clones containing BG-1 16S rRNA genes were obtained by PCR screening (S1 Table). The two fosmid clones harboring the BG-1 16S rRNA gene and the 11 selected positive fosmid clones harboring GH genes were sequenced and assembled into fosmid contigs using the Newbler software (S1 File). The fosmid contigs were annotated in the same method as the GH-containing contigs and deposited in GenBank under accession numbers KJ797017—KJ797027.

The depth of contigs in the metagenomic data was calculated to weight the distribution of these contigs and the methods are described in S1 File [36].

### Expression of 6 recovered GH5 family genes in *E*. *coli*

Six recovered GH5 genes (Cel1–Cel6) with full-length sequence were expressed in *E*. *coli* (S2 and S3 Tables) and the purified enzymes were subjected to testing for lignocellulase activity on the substrates carboxymethyl-cellulose (CMC, Sigma, St. Louis, MO, USA), locust bean gum (Sigma, St. Louis, MO, USA), xylan from beechwood (Sigma, St. Louis, MO, USA), and p-nitrophenyl-D-cellobioside (pNPC, Sigma, St. Louis, MO, USA). The six GH5 genes were assigned to different GH5 subfamilies according to phylogenetic analysis based on their closest GH5 gene relatives. Gene sequences, gene expression, enzymatic analyses, phylogenetic analysis, and signal peptide prediction details for the GH5 genes are described in S1 File.

## Results

### 16S rRNA gene based analysis reveals the microbial diversity in biogas digesters

The 16S rRNA genes amplified from DNA isolated from biogas digesters Z7 and Z8 were sequenced and subjected to phylogenetic analysis and diversity estimation. Using a similarity threshold of ≥99% for operational taxonomic unit (OTU) determination, there were 225 and 151 bacterial OTUs, and 14 and 8 archaeal OTUs observed for Z7 and Z8, respectively (S4 Table; S2 File and S3 File). The Good coverage index indicated that more than 84% of bacterial diversity and 95% of archaeal diversity was sampled from the two communities (S4 Table).

*Firmicutes* was the largest bacterial phylum (72.77% of Z7 and 48.68% of Z8), followed by the *Bacteroidetes* (20.77% of Z7 and 37.42% of Z8) and the *Proteobacteria* (4.26% of Z7 and 6.79% of Z8) in the 16S rRNA clone libraries (S5 Table; S2a Fig). Of the 339 bacterial OTUs identified from Z7 and Z8, 216 OTUs were singletons, indicating that most bacteria in the biogas digesters were present in low abundance (S2 File). Only 13 OTUs had ≥ 97% identity to cultured bacteria (i.e., type strains), though most bacterial OTUs matched sequences previously identified in microbial communities related to anaerobic fermentation systems (S2 File).

Twenty-six dominant bacterial OTUs represented by more than 10 sequences in clone libraries from both biogas digesters are summarized in Table 1. None of the dominant bacterial OTUs shared more than 97% identity with any cultured bacterial type strains. However, OTU158 and OTU98, which were dominant in Z7, were related to the cellulolytic strain *Clostridium straminisolvens* (T) CSK1 [37] with 94.76% and 92.29% identities, respectively. The *C*. *straminisolvens* (T) CSK1 16S rRNA gene shares 96.2% identity with that of the cellulolytic bacterium, *C*. *thermocellum*. The *C*. *thermocellum*-like bacteria, OTU158, OTU98, *C*. *straminisolvens*, and *C*. *thermocellum* clustered together in the phylogenetic tree (S3 Fig). OTU48 (hereafter named BG-1) shared 99.93% identity with one uncultured cellulolytic bacterium whose function was previously identified by FISH and DNA-SIP analyses[10]. BG-1 was taxonomically assigned to *Clostridia* group 4, and determined to be distinctively different from the *C*. *thermocellum*-like bacteria [10]. Further phylogenetic analysis of the BG-1 16S rRNA sequence indicated that BG-1 reveals a new order in the class *Clostridia* (S3 Fig). Besides, it is difficult to determine the functions of the other dominant OTUs due to the lack of close relatives with known functional information, or in some cases, the lack of any close phylogenetic relatives.

**Table 1:** The most dominant OTUs and their compositions in the bacterial 16S rRNA gene clone libraries and metagenomes of Z7 and Z8.

OTU_ID	Phylum	Nearest bacteria; Accession number (Identity^1^, %)	Nearest type strain; Accession number (Identity^1^, %)	Z7_ PCR^2^	Z8_ PCR^2^	Z7_ META^3^	Z8_ META^3^
OTU4*	Bacteroidetes	uncultured bacterium, DGGE band B5; EF597508 (100)	*Alistipes shahii* (T) WAL 8301; AY974072 (83.72)	4.00%	12.91%	0.95%	5.84%
OTU48*	Firmicutes	uncultured bacterium,C35_D63_H_B_F12; EF559123 (100)	*Desulfosporosinus auripigmenti* (T); AJ493051 (85.44)	11.10%	2.98%	7.59%	3.65%
OTU178*	Firmicutes	uncultured bacterium, 194BF26; AB330672 (100)	*Pelotomaculum thermopropionicum* (T) SI; AB035723 (85.42)	8.13%	0.00%	7.28%	0.00%
OTU3*	Firmicutes	uncultured bacterium, AA39;EF016592 (98.62)	*Bacillus nealsonii* (T) FO-092; AF234863 (86.93)	0.26%	8.44%	2.37%	4.50%
OTU33*	Firmicutes	uncultured bacterium, ATB-KS-1506; EF686979 (99.87)	*Caloranaerobacter azorensis* (T) MV1087; AJ272422 (86.78)	4.90%	1.66%	1.74%	1.34%
OTU19	Bacteroidetes	uncultured Bacteroidetes bacterium, B9; EU551120 (99.65)	*Alkaliflexus imshenetskii* (T) Z-7010; AJ784993 (89.74)	2.19%	4.30%	0.32%	2.68%
OTU15*	Bacteroidetes	uncultured bacterium, PIST-AFB12; AM982543 (99.71)	*Rikenella microfusus* (T); L16498 (84.33)	0.00%	6.95%	0.16%	3.77%
OTU20	Bacteroidetes	uncultured bacterium, ATB-KS-1507; EF686980 (100)	*Bacteroides eggerthii* (T); L16485 (85.90)	2.71%	2.65%	2.53%	4.38%
OTU158*	Firmicutes	uncultured bacterium, ATB-KS-1450; EF686967 (99.87)	*Clostridium straminisolvens* (T) CSK1; AB125279 (94.76)	4.52%	0.00%	4.11%	0.00%
OTU166*	Firmicutes	uncultured bacterium, DGGE band W1-A; AM932206 (94.78)	*Desulfotomaculum luciae* (T) SLT; AF069293 (85.81)	4.00%	0.00%	3.32%	0.24%
OTU38*	Tenericutes	uncultured bacterium, C35_D7_L_B_E10; EF559103 (99.13)	*Acholeplasma palmae* (T); J233 (89.80)	0.13%	4.47%	0.63%	1.95%
OTU41*	Firmicutes	uncultured bacterium, AA39;EF016592 (96.53)	*Moorella glycerini* (T) YS6; U82327 (86.19)	0.13%	4.14%	1.74%	2.19%
OTU172*	Bacteroidetes	uncultured bacterium, ATB-KS-1932; EF686990 (99.87)	*Cytophaga fermentans* (T); M58766 (85.79)	3.23%	0.00%	2.06%	0.12%
OTU5*	Bacteroidetes	uncultured bacterium, ATB-KS-1940; EF686992(100)	*Cytophaga fermentans* (T); M58766 (85.20)	0.26%	3.81%	0.16%	1.58%
OTU292*	Firmicutes	uncultured bacterium, 135BF32;AB330651 (99.60)	*Desulfotomaculum luciae* (T) SLT; AF069293 (87.11)	2.45%	0.00%	6.17%	0.00%
OTU30	Firmicutes	uncultured bacterium; DGGE band W1-A; AM932206 (95.33)	*Moorella glycerini* (T) YS6; U82327 (86.23)	1.94%	0.50%	2.06%	1.58%
OTU17*	Proteobacteria	uncultured bacterium, A35_D28_H_B_A04; EF559229 (99.42)	*Pseudomonas pseudoalcaligenes* (T); Z76675 (93.70)	0.39%	2.48%	0.16%	2.19%
OTU156*	Firmicutes	uncultured bacterium, B55_K_B_E03; DQ887967 (99.78)	*Clostridium thermosuccinogenes* (T); Y18180 (93.74)	2.19%	0.00%	5.70%	0.00%
OTU162*	Bacteroidetes	uncultured eubacterium, WJGRT-163; AF175658 (99.78)	*Petrimonas sulfuriphila* (T) BN3; AY570690 (81.46)	1.94%	0.00%	0.79%	0.00%
OTU185*	Firmicutes	uncultured bacterium, 135BF29; AB330648 (99.00)	*Ruminococcus callidus* (T); L76596 (91.93)	1.94%	0.00%	0.63%	0.00%
OTU98	Firmicutes	uncultured bacterium; 135BF26; AB330645 (99.80)	*Clostridium straminisolvens* (T) CSK1; AB125279 (92.29)	1.68%	0.33%	2.37%	0.12%
OTU35	Firmicutes	uncultured bacterium, 135BF21; AB330640 (99.80)	*Clostridium jejuense* (T) HY-35-12; AY494606 (92.31)	0.39%	1.32%	0.79%	1.09%
OTU6	Verrucomicrobia	uncultured bacterium, GZKB73; AJ853567 (99.09)	*Candidatus Xiphinematobacter rivesi* (T); AF217461 (79.94)	0.39%	1.32%	0.47%	1.46%
OTU309	Firmicutes	uncultured bacterium, ATB-KS-1443; EF686964 (99.74)	*Desulfotomaculum luciae* (T) SLT; AF069293 (87.27)	1.29%	0.00%	2.22%	0.36%

^1^The identity of 16S rRNA genes between representative sequence of each dominant OTU and their nearest bacteria or type strains.

^2^Z7_PCR and Z8_PCR represent the proportion of each OTU in the 16S rRNA gene clone library of Z7 and Z8.

^3^Z7_META and Z8_META represent the proportion of each OTU in all the 16S rRNA genes recovered from Z7 and Z8 metagenomic data.

* Significant differential OTUs between Z7 and Z8 16S rRNA clone libraries were identified and filtered (q-value, <0.001) using STAMP (permutation test).

The phylogenetic group distributions were compared via 16S rRNA gene clone libraries and 16S rRNA gene reads extracted from the metagenome, indicating that the phylogenetic diversities of 16S rRNA clone libraries and metagenomic 16S rRNA gene datasets in Z7 or Z8 had consistent patterns (S5 Table; S2 Fig). When comparing them on the species level, most of the bacterial PCR-based 16S rRNA gene sequences (93.6%) had counterparts in the metagenomic 16S rRNA gene reads, with a minimum alignment length of 60 nucleotides and 98–100% sequence identity, and *vice versa* (85.6%). In all, the consistency of the phylogenetic distribution which was revealed by 16S rRNA gene analysis derived from PCR amplicons and 454 reads of the metagenome gene suggested that most of the microbial diversity in Z7 or Z8 was included in the metagenomic data (S5 Table; S2 Fig).

### Short metagenomic reads further reveal *C*. *thermocellum*-like bacteria were dominant in Z7 and Z8

A total of 519 015 and 668 860 reads were generated from the pyrosequencing of Z7 and Z8, respectively (S6 Table). Here, 3965 contigs ≥ 1 kb in length (average size = 1742 bp), summing up to 6.91 Mbp, were assembled from 174 221 metagenomic reads by the Newbler software; the largest contig was 22 059 bp. As only a small portion of the short metagenomic reads were assembled (14.67% of all Z7 and Z8 metagenomic reads), the short metagenomic reads were used in BLAST annotation, with hits from the NR, COG/KEGG, and Ribosomal Database Project (RDP) databases. In total, 68% of the metagenomic reads were assigned to NCBI taxonomy at various levels (S6 Table), and 40% of the reads were assigned at the family level, a reliable level as it was demonstrated by MEGAN analysis (S4a Fig) [33]. Almost all of the EGTs belonged to bacteria or archaea, only about 0.6% were assigned to eukaryotes, including fungi. A total of 44 phyla were identified, and *Firmicutes*, *Bacteroidetes*, *Euryarchaeota* and *Proteobacteria* were the dominant phyla in both samples (S4b Fig), which was consistent with the dominant phyla in 16S rRNA gene analysis (S5 Table; S2a Fig). Approximately 30% of the EGTs from the metagenomes were assigned species-level taxonomy (S4a Fig) and most of the dominant species (> 1000 reads) were common in both Z7 and Z8 (S7 Table). Three cellulolytic bacterial species (*C*. *thermocellum*, *C*. *cellulolyticum*, and *Caldicellulosiruptorsaccharolyticus*) were dominant, and many metagenomic reads were assigned to the *C*. *thermocellum* genome (S7 Table).

### Diverse GH genes and other related genes involved in lignocellulose degradation were revealed through annotation of short metagenomic reads

**Fig 1:**
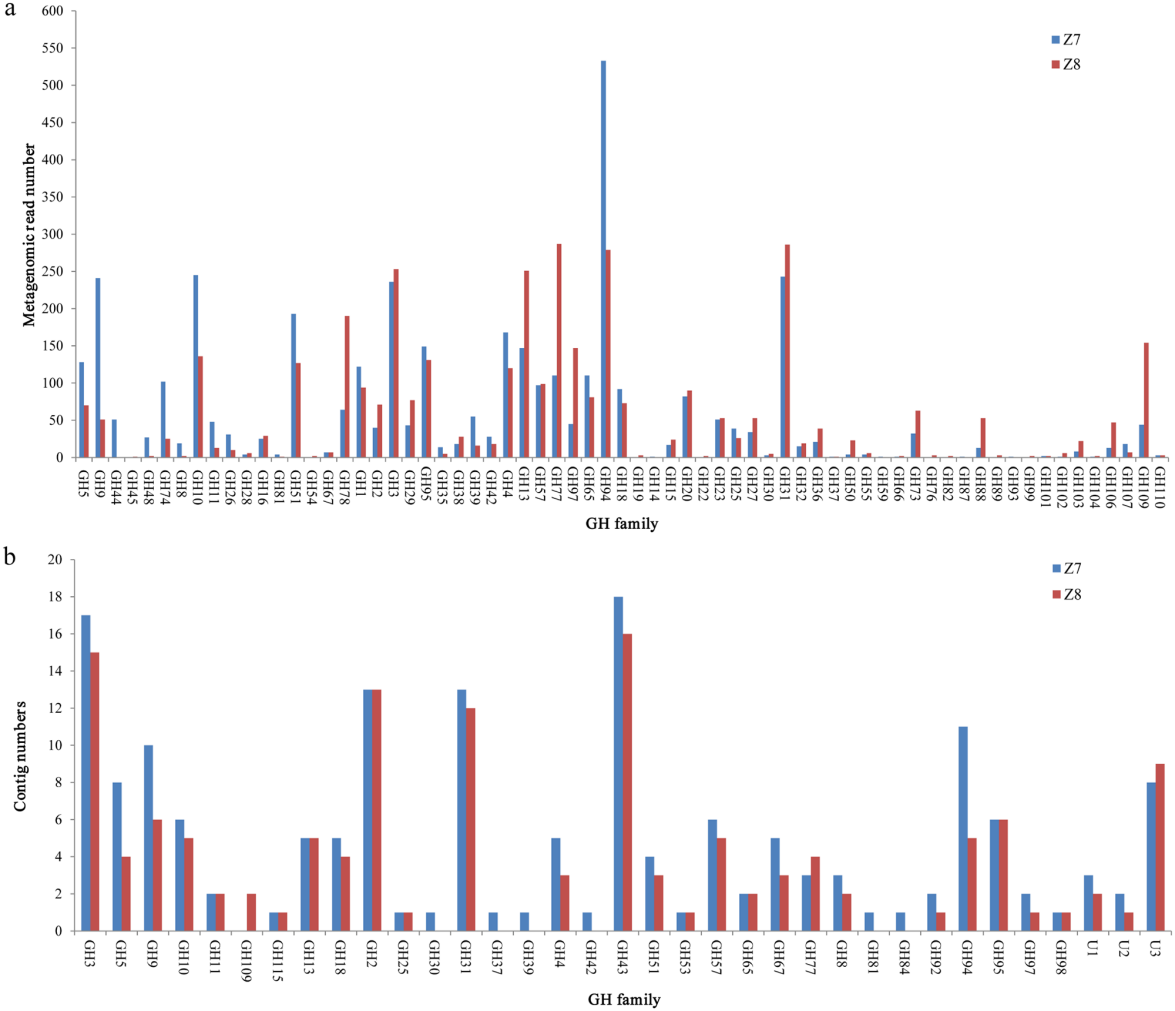
The number of metagenomic reads and contigs assigned to different GH family. (a) The number of metagenomic reads assigned to the encoding genes of different GH family. (b) The number of contigs recovered from the metagenomic short reads by the refined assembly approach assigned to the encoding genes of different GH family. U1, U2 and U3 refer to unclassified α-L-arabinofuranosidase, unclassified xylosidase and unclassified α-amylase, respectively.

COG and KO categories related to GH and associated proteins involved in the hydrolysis of carbohydrates such as starch, cellulose, and hemicellulose, were analyzed in Z7 and Z8. There were 3850 EGTs in Z7 that were assigned to 59 GH families and 3703 EGTs in Z8 assigned to 62 GH families, representing a rich diversity of putative lignocellulase and other GH genes (Fig 1a; S8 Table). Among them, the EGTs of lignocellulase genes were distributed mainly among the GH1, GH3, GH5, GH9, GH10, and GH11 families (Fig 1a; S8 Table).

Carbohydrate-binding modules appended with GHs play a critical role in lignocellulose degradation [38], and 603 EGTs assigned to 16 CBM families were identified (S9 Table). CBM6 and CBM36, which often link to the xylanase catalytic module and have an affinity for xylan, were dominant in the two metagenomes. In addition, CBM4, CBM6, and CBM9, which have affinity for amorphous cellulose, were also abundant. Similarly, diverse EGTs associated with cellulosome-associated regions—such as cohesin and dockerin domains—were also identified, confirming that *C*. *thermocellum-*like bacteria were dominant in the biogas digesters (S10 Table).

### Recovery of dominant GH-containing contigs using the refined assembly approach and validation of gene assemblies by PCR amplification and sequencing

The refined assembly approach developed in this study was used to recover dominant lignocellulase genes (GH1, GH3, GH5, GH9, GH10, and GH11) from the two biogas digesters. A total of 44,477 metagenomic reads were HSP hits and were retrieved from the metagenomic data. Using the refined assembly approach, these metagenomic reads were assembled into 7438 contigs; 219 contigs had lengths ≥ 1 kb (S11 Table) and among these, 163 contigs contained at least one catalytic module belonging to a GH family (Fig 1b; S12 Table). Moreover, a comparison of GH-containing contigs assembled by the refined approach with the corresponding contigs assembled by the Newbler software revealed that the refined approach resulted in recovery of larger contigs (S12 Table). In total, 181 catalytic modules belonging to more than 32 GH families were recovered, including: 17 GH3 genes, 8 GH5 genes, 11 GH9 genes, 6 GH10 genes, and 2 GH11 genes, as well as many GH genes related to diverse hemicellulases, such as α-arabinofuranosidases (GH43, GH51, and unclassified arabinofuranosidases), α-glucuronidases (GH67 and GH115) and α-xylosidases (GH31, GH43, and unclassified α-xylosidase) (Fig 1b; S12 Table). However, no GH1-containing contigs ≥ 1 kb in length were obtained.

To determine whether the assembled GH-containing contigs were present in the biogas digesters, 50 pairs of contig-specific primers were designed from 50 randomly selected GH-containing contigs and used to amplify corresponding fragments from the metagenomic DNA sample isolated from Z7 as the template (S1 and S12 Tables). PCR fragments of the predicted sizes were obtained for all 50 contigs (100%), and their sequences were nearly identical to their corresponding target regions on the 50 assembled contigs (≥ 95% sequencing identity). These results confirmed that the recovered GH genes in the contigs assembled from metagenomic reads represented authentic genes in the metagenome of the biogas digesters.

### Sequencing and analysis of fosmid clones screened from the Z7 fosmid library provide insights into GHs of dominant cellulolytic bacteria

To obtain more genomic information of the dominant cellulolytic bacteria, several contig-specific primers for the GH-containing contigs and the BG-1 16S rRNA gene were used to perform sequence-based screening of a previously constructed fosmid library [16]. After screening, 11 fosmid clones harboring GH-containing contigs (mainly GH5 and GH9 genes) and two fosmid clones harboring BG-1 16S rRNA genes were subjected to sequencing.

**Fig 2:**
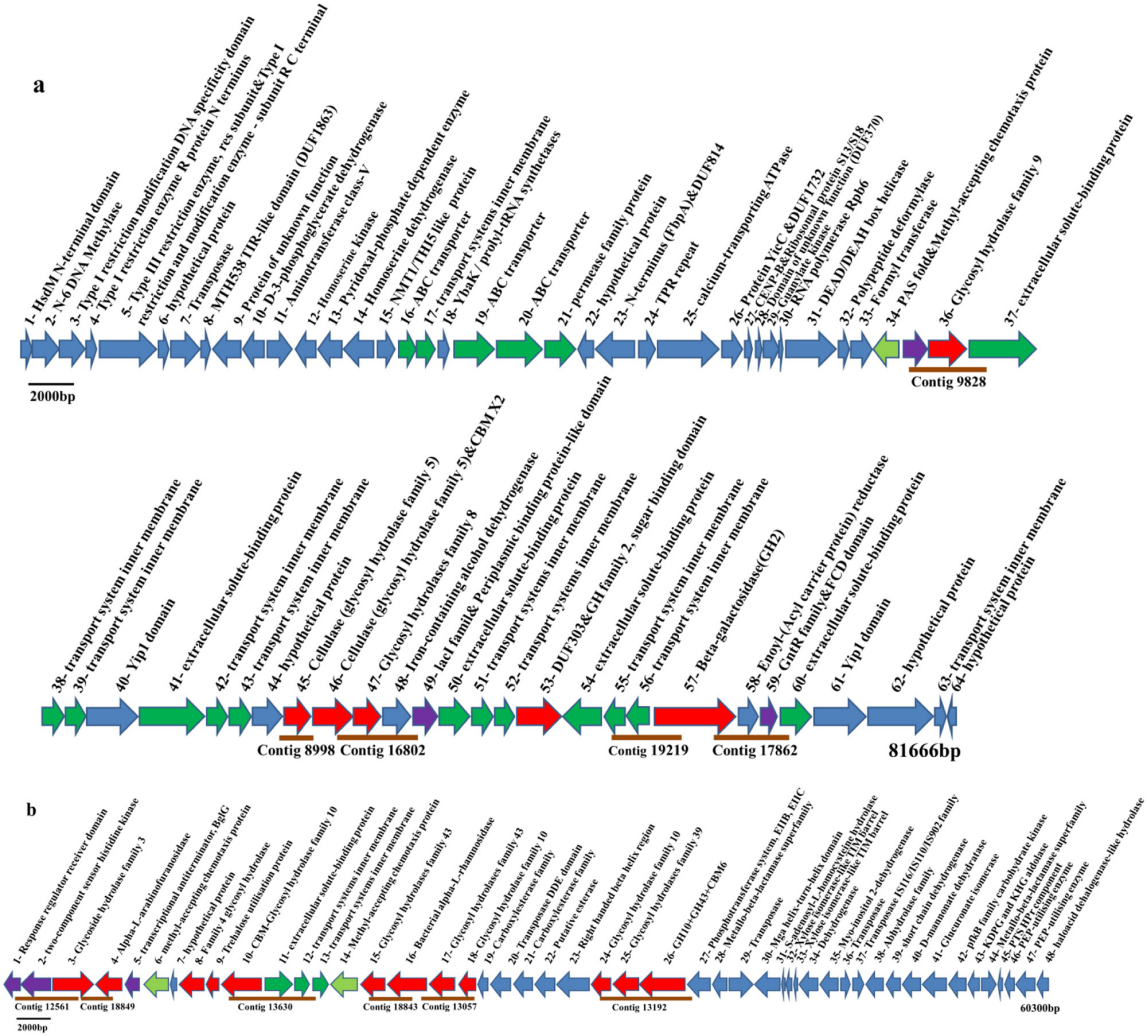
Gene organizations on the contigs FC1 and FC2 recovered from biogas digester. (a) Contig FC1: a fosmid contig harboring GH genes assembled from Fosmid 76N21 and Fosmid 86J18. (b) Contig FC2: a fosmid contig harboring GH genes assembled from Fosmid 76E20 and Fosmid 93M19. The scale bars display a length of 2000bp. The GH-containing contigs recovered with the refined assembly approach in the contigs of FC1 and FC2 are showed in parallel. Red, GH genes; Purple, genes encoding regulatory proteins; Light green, genes encoding PAS folds and methyl-accepting chemotaxis proteins (MCP); Dark green, genes encoding proteins related with transport systems; Blue, genes encoding proteins with other functions or hypothetical proteins without known domains.

Of the 13 sequenced fosmid clones, only one clone was not fully recovered (Fosmid 255O19), due to assembly errors resulting from repeated cohesin domain sequences (Table 2). Assembly of the 13 fosmid clones resulted in 11 fosmid contigs and 0.45 Mbp of DNA sequence (Table 2). Fosmid contig FC3, 51 kb in length, assembled from two fosmid clones harboring BG-1 16S rRNA genes, contained no GH genes (Table 2). The gene structures of the 11 fosmid contigs are shown in Fig 2 and S5 Fig. Several GH family genes related to cellulose or hemicellulose degradation formed GH gene clusters in some fosmid contigs, including FC1 and FC2. Various AraC, LacI, BglG, or GntR family-like transcriptional regulators were detected in clusters containing GH family genes in the fosmid contigs, suggesting that these negative regulators might regulate GH gene expression [39, 40]. Homologues of transporters, such as ABC transporters and *ton*B-dependent receptors, which may be used in lignocellulose degradation processes [21], were linked with lignocellulase genes in FC1, FC2, and FC4 (Fig 2; S5 Fig). In addition, dockerin and cohesin domains were found in FC10R, linked with one GH9 gene (S5 Fig).

**Table 2:** Eleven fosmid contigs assembled from sequences of fosmid clones harboring GH-containing contigs or BG-1 16S rRNA genes.

Fosmid contigs ID	Sequences used for assembly	Length(bp)	Number of GH-containing contigs^1^	Metagenomic reads number in Z7	Metagenomic reads number in Z8	Depth	Taxonomic binned by PhyloPethiaS
FC1	Fosmid76N21;Fosmid86J18	81666	5	1139	314	4.51	BG-1
FC2	Fosmid76E20;Fosmid93M19	60300	6	1011	274	5.39	BG-1
FC3	Fosmid81E17; Fosmid83P13	51420	0	856	314	5.76	BG-1^2^
FC4	Fosmid86J4	42441	1	182	966	6.87	*Clostridia*
FC5	Fosmid93D17	40839	2	187	211	2.47	Bacteria
FC6	Fosmid251K21	40480	1	199	900	6.89	*Clostridia*
FC7	Fosmid276P23	40345	1	139	0	0.87	*Clostridia*
FC8	Fosmid82I22	39981	1	374	0	2.37	*Clostridia*
FC9	Fosmid88I5	35121	2	508	120	4.53	*Clostridia*
FC10R	Fosmid255O19	19704	1	585	2	7.54	*Clostridia*
FC10F	Fosmid255O19	14283	1	Unknown^3^	Unknown^3^	Unknown^3^	*Clostridia*

^1^Number of GH-containing contigs harbored in each fosmid contig.

^2^Due to contig FC3 contained BG-1 16S rRNA gene, it was assigned to BG-1.

^3^Due to presence of conserved cohesin domain in FC10F, it was unable to accurately bin metagenomic reads to FC10F and calculated depth of FC10F.

### BG-1 genomic information recovered by PhyloPythiaS analysis

Of the 10 GH-containing fosmid contigs, PhyloPythiaS analysis using FC3 as the sample-specific model, supported the assignment of FC1 and FC2 to BG-1, while the other fosmid contigs were assigned either to the order *Clostridia* or to unknown bacteria (Table 2) [24]. In addition, another 214 contigs ≥ 1 kb in length assembled from Z7 and Z8 metagenomic reads by the Newbler software, were also assigned to BG-1 (S13 Table). The depth of 3 fosmid contigs (FC1, FC2 and FC3) and most other contigs assigned to BG-1 was similar. Based on these analyses, ~ 0.53 Mbp of BG-1 genomic sequences were obtained, suggesting that BG-1 possesses at least 21 genes related to lignocellulose degradation. Specifically, there were 12 different lignocellulase genes, including GH3, GH4, GH10, GH39, GH43, as well as genes encoding α-L-rhamnosidase and α-arabinofuranosidase genes in FC2 (Fig 2b). In addition, FC1 possesses six GH genes, including two GH2 genes, one GH8 gene, two GH5 genes, and one GH9 gene (Fig 2a). According to the characterized proteins in the CAZy database, these lignocellulases are believed to degrade different components of the plant cell wall. No genes encoding cellulosomal components were detected in the GH-containing contigs or fosmid contigs assigned to BG-1, further testifying that BG-1 differs from the *C*. *thermocellum*-like bacteria.

In addition to the lignocellulose degradation potential, the molecular basis for the transmembrane import of oligosaccharides into the cytoplasm of BG-1 was emphasized by identification of four predicted ABC-type transporters within the FC1 gene cluster. Significantly, there were three extracellular solute-binding proteins (SBP family 1; gene 37, gene 50, and gene 60) among the ABC-type transporters in FC1, which are predicted to bind oligosaccharides and facilitate interactions with proximal permeases in the bacterial cell membrane [41]. Moreover, one ABC transporter (gene 11 to gene 13) predicted to transport sugar was also present in FC2. Additionally, several bacterial transmembrane sensor proteins, including two *lac*I family-like regulators (gene 35 and gene 49), one GntR family-like regulator (gene 59), and one methyl-accepting chemotaxis protein (MCP, gene 34), were present in FC1 [42]. One predicted operon comprised gene 35–gene 47, which might be co-expressed in BG-1 (Fig 2a). One BglG-like regulator linked with GH genes, and two MCP signaling domains (gene 6 and gene 14), were present in FC2 (Fig 2b).

### Expression, purification, and characterization of six GH5 genes in *E*. *coli*

The GH5 gene family is one of the largest families in the CAZy database and ~80% of GH5 genes can be classified into 51 distinct subfamilies [43]. GH5 was also one of the most dominant GH families in the biogas digesters [32]. Six full-length GH5 genes were recovered from the fosmid contigs; three of these (Cel2, Cel5 and Cel6) belonged to BG-1. Phylogenetic analysis indicated that three of the genes (Cel2, Cel4, and Cel6) could be assigned to subfamily GH5_4, one (Cel5) to subfamily GH5_10, while the remaining two genes (Cel1 and Cel3) could not be assigned to any subfamily (Table 3; S6 Fig). Among the six genes, Cel2, Cel4, Cel5, and Cel6 were predicted to have signal peptides.

**Table 3:** The characterization of six GH5 genes expressed in *E*.*coli*.

Gene ID	Signal peptides	Nearest neighbor; Accession number (Identity, %)^1^	GH5 subfamily	Endoglucanase^2^	Mannanase^2^	Xylanase^2^	Exocellulase^2^
Cel1	No	*Ruminococcus champanellensis* 18P13; CBL16471 (50)	unclassified	ND^3^	1255±29.9	7.56±0.37	ND^3^
Cel2	Yes	*Eubacterium siraeum* 70/3; CBK96866 (50)	GH5_4	ND^3^	16.67±0.86	12.4±0.71	ND^3^
Cel3	No	*Cytophaga hutchinsonii* ATCC 33406; YP_678451 (49)	unclassified	ND^3^	ND^3^	ND^3^	Low^4^
Cel4	Yes	Uncultured bacterium; AEV59736 (66)	GH5_4	ND^3^	Low^4^	14.81±0.22	Low^4^
Cel5^5^	Yes	*Spirochaeta thermophila* DSM 6192; YP_003873341 (55)	GH5_10	Low^4^	85.13±3.34	1.03±0.06	Low^4^
Cel6^5^	Yes	*Mahella australiensis* 50–1 BON; YP_004463133 (54)	GH5_4	Low^4^	ND^3^	Low^4^	ND^3^

^1^Nearest neighbor represented nearest protein sequence for each GH5 genes.

^2^All activity assays were performed at pH7.4 and 50°C. The substrates used to determine activities of endoglucanase, mannanase, xylanase and exocellulase were CMC, locust bean gum, xylan (beechwood) and pNPC. The unit of enzyme activity is U/mg protein.

^3^Activity was not detected.

^4^Activity was less than 1 U/mg protein.

^5^Cel5 and Cel6 were also mentioned as man1 and en2 in elsewhere (Yan *et al*., 2013), but were not expressed before.

The six GH5 genes were expressed in *E*. *coli* BL21 and the purified proteins were tested for enzymatic activity on CMC, locust bean gum, xylan (beechwood), and pNPC. The results indicated that most of the GH5 proteins have a wide substrate range (Table 3). Remarkably, Cel5 showed activity in all tests, including endoglucanase, xylanase, mannanase, and exocellulase activities. Cel1, one of the unclassified GH5 proteins, exhibited mannanase activity of 1255 U/mg protein, which is one of the highest mannanase activities ever reported in the BRENDA database [44]. Another GH5 unclassified protein, Cel3, exhibited weak exocellulase activity (Table 3).

## Discussion

Although more than half dominant OTUs listed in Table 1 were shared by Z7 and Z8, their distributions in these two reactors were significantly different. For example, OTU48 (BG-1) was the dominant cellulolytic species in Z7 and Z8, its distribution was much more abundant in Z7 than Z8 (Table 1). Besides, metagenomic taxonomy analysis by MEGAN also suggested the abundance of cellulolytic species was significantly higher in Z7 than Z8 (S7 Table). Because the additional rice straw feeding in Z7 was the exclusive different operating condition for the two biogas digesters, feeding rice straw might contribute to the enrichment of cellulolytic species in Z7. However, more parallel experiments were necessary to support this hypothesis. In addition, MEGAN analysis also revealed the domain methangen, *Methanoculleus marisnigri*, was much more abundant in Z8 than Z7 (S7 Table). Though the chemical analysis of the slurry in these two biogas digesters were similar, the methane yield of Z8 was much lower than that of Z7 (S14 Table). These results suggested that the relative abundances between cellulolytic bacteria, methanogens and other functional groups might be important to achieve high methane yield, which would be investigated in the future.

Using the refined assembly approach developed in this study, 163 GH-containing contigs were successfully recovered from the short reads of the low-coverage metagenomic data (S12 Table). The 50 assembled GH-containing contigs selected at random were verified to be correct, suggesting that the refined approach can be used in the recovery of lignocellulase genes or other functional genes from similar short read metagenomic datasets generated with second-generation sequencing technology, such as Solexa technology. Though only gene sequences predicted as dominant lignocellulase by short 454 reads from the GH1, GH3, GH5, GH9, GH10, and GH11 families in the CAZy database were used as reference genes, 122 untargeted GH-containing contigs were recovered in addition to the 41 targeted contigs containing GH3, GH5, GH9, GH10, or GH11 family genes.This may be due to GH genes are ambiguous nonhomologous [15] or the parameters in the refined assembly approach used to retrieve metagenomic GH reads were loose. However, no GH1 genes were identified in the GH-containing contigs and fosmid contigs, implying that the GH1 family genes may have been ambiguously annotated and were not associated with the dominant cellulolytic bacteria [15]. Diverse GH genes and other related genes involved in lignocellulose degradation (CBM and cellulosomal genes) were recovered in the GH-containing contigs and fosmid contigs, suggesting that cellulolytic bacteria harboring these GH genes can efficiently degrade lignocellulose. The free enzyme system, the cellulosome system, and the predicted SusC (*ton*B)/SusD-mediated mechanism are the three known mechanisms used by microorganisms to efficiently degrade lignocellulose [20, 21, 38]. Because no processive cellulase genes (mainly distributed in the GH6, GH7, and GH48 families) were found in the biogas digesters, the free enzyme system might not have been present [38]. Cellulosomal genes were linked with GH genes in several recovered GH-containing contigs and fosmid contigs, and a *ton*B-dependent receptor gene and a GH9 gene were clustered in FC5, implying that the cellulosome system common in *C*. *thermocellum*-like bacteria as well as the SusC/SusD-mediated mechanism are present in the biogas digesters. Theoretically, the metagenomic data is a random sample of the entire microbial community; thus metagenomic reads containing GH gene sequences from the dominant cellulolytic bacteria should be abundant and easily assembled into large contigs.

BG-1 was first identified as an uncultured cellulolytic bacterium through DNA-SIP and FISH observations of a landfill leachate-derived mesophilic anaerobic digester in France [10], and has since been identified in other biogas digesters [45, 46]. The analysis of 16S rRNA genes obtained from the clone libraries and metagenomic reads from our two biogas digesters indicated that BG-1 was one of the dominant cellulolytic bacteria (Table 1). However, as no genome information is available for BG-1 or its closest phylogenetic relatives (S3 Fig), and more than 70% of the metagenomic reads could not be taxonomically classified at the species level (S4a Fig), it was impossible to evaluate the abundance of BG-1 in our biogas digesters or obtain genomic information on BG-1 using MEGAN analysis.

**Fig 3:**
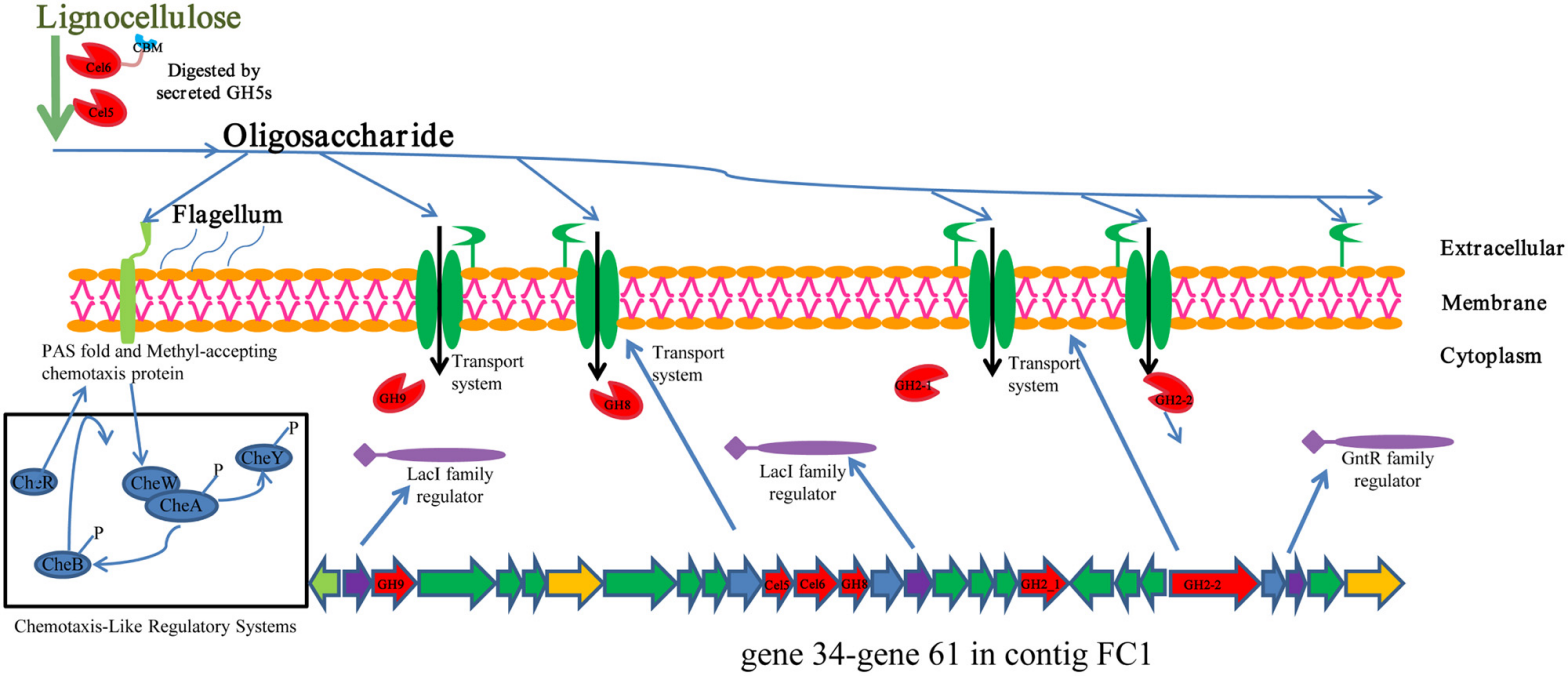
The hypothetical lignocellulose degradation mechanism predicted from gene 34 to gene 61 in contig FC1 of BG-1. The 28 genes were predicted to encode two LacI family transcriptional regulators (purple), one GntR family transcriptional regulator (purple), PAS fold and methyl-accepting chemotaxis protein (light green), proteins of four different ABC transport system (green), six GHs (2GH5, Cel5 and Cel6; one GH9; one GH8; 2 GH2, GH2_1 and GH2_2) (red) and other functional proteins. The chemotaxis-like regulatory system described in the black box was inferred from other BG-1 genome information.

Using PhyloPythiaS analysis, ~0.53 Mbp of BG-1 genomic sequences were obtained (Table 2; S13 Table). Based on the assembled genome sequences, some aspects of the lignocellulose degradation mechanism used by BG-1 could be deduced. Though BG-1 possesses diverse GH genes and may specialize in lignocellulose degradation, no genes encoding cellulosomal components or SusC/SusD were assigned to BG-1 by PhyloPythiaS analysis. Based on the gene structures of FC1 and FC2, and using FC1 as example, we propose here a possible novel lignocellulose degradation mechanism that might be used by BG-1 (Fig 3) [39, 40]. At first, all GH genes are expressed at low-levels and small amounts of the two GH5 proteins, Cel5 and Cel6 (Table 3), are secreted into the surrounding environment. When BG-1 is exposed to lignocellulose, it is hydrolyzed into oligosaccharides by the secreted GH5 proteins. Next, the PAS-fold domain and MCP signaling domain [42] detect rising levels of oligosaccharides released by GH5s from the extracellular environment and modulate BG-1 towards a lignocellulose-present environment [47]. The oligosaccharides are imported into cytoplasm by ABC transporters and hydrolyzed into simple sugars by other GH proteins in the cytoplasm, to be used for bacterial cell growth. Some of the simple sugars bind to the sugar-binding domain of the negative regulators, such as the lacI and GntR family-like regulators. The regulator-sugar complex is then released from the promoter region in the GH gene cluster, resulting in high expression of the GHs and ABC transporters identified in FC1. High expression of the GH proteins and ABC transporters results in efficient hydrolysis and utilization of lignocellulose by BG-1. As several genes are located in the same operon(such as gene 35–gene 47), these genes might be regulated by the same negative regulators and therefore co-transcribed [39, 40]. When the expression levels of negative regulators overcome the intracellular sugar levels (i.e., when the lignocellulose in the vicinity of BG-1 is consumed), transcription of the GH gene cluster is severely down-regulated and returns to the low-level expression mode. As BG-1 was the predominant cellulolytic bacterium identified, this newly proposed mechanism suggests a possible alternative lignocellulose degradation mechanism and might be one of the dominant lignocellulose degradation mechanisms in these two biogas digesters. However, this possible mechanism is mainly speculative derived from the part genomic information of BG-1, and more experimental verifications are required in future studies.

Two further dominant OTUs in Z7 (OTU158 and OTU98) were closely related to the cellulolytic bacterium, *C*. *straminisolvens* (T) CSK1, a *C*. *thermocellum*-like bacterium (Table 1). PCR-based 16S rRNA gene analysis identified only five singleton OTUs in Z7 that were closely related to *C*. *thermocellum*, with shared identities of 86–89%. However, MEGAN analysis indicated that *C*. *thermocellum* was the dominant cellulolytic bacterium, and many metagenomic reads were assigned to the *C*. *thermocellum* genome (S7 Table). As the genome of *C*. *straminisolvens* (T) has not yet been available in our MEGAN analysis, and *C*. *straminisolvens* (T) is phylogenetically similar to *C*. *thermocellum*, we speculate that MEGAN might have assigned most reads from OTU158 and OTU98 to the *C*. *thermocellum* genome. This speculation is supported by the fact that the compositions of reads from Z7 and Z8 that were assigned to C. *thermocellum* (3.54% and 0.36%, respectively) based on MEGAN analysis were consistent with the compositions of OTU158 and OTU98 (6.81% and 0.33%, respectively) in Z7 and Z8 16S rRNA gene clone libraries (Table 1; S7 Table).

Many different activities relevant to lignocellulose degradation have been identified in the GH5 family [43]. The six GH5 family genes assembled, expressed, and characterized in this study exhibited diverse lignocellulase activities. Although Cel2 and Cel4 were predicted to be members of subfamily GH5_4, which has been reported to exhibit only three different activities (endo-β-1, 4-glucanases, licheninases, and xylanases), we determined that Cel2 and Cel4 exhibited both mannanase activity and xylanase activity. In addition, Cel4 demonstrated exocellulase activity (Table 3). Cel5 was a member of subfamily GH5_10, a subfamily which has been reported to possess only mannanase activity [43]. However, Cel5 demonstrated not only mannanase activity, but also activities toward xylan and pNPC. This suggests that GH5_10 may be a subfamily with polyspecific activity (Table 3). Based on enzymatic analysis of the six GH5 proteins, the activity spectra of subfamilies GH5_4 and GH5_10 have been expanded. In future studies, more substrates should be used in the characterization of novel GH5 proteins. As a phylogenetic clade with five or more GH5 family genes in the public protein databases obtained from different microorganisms can be defined as a new subfamily of the GH5 family [43]. Cel1 and Cel3 and their clades can be seen as two new GH5 subfamily candidates (S6 Fig). Therefore, the enzymatic analysis of the six GH5 proteins has expanded the activity spectrum of subfamilies GH5_4 and GH5_10, and provided two new GH5 subfamily candidates with characterized proteins, further demonstrating that the recovered GH-containing contigs and fosmid contigs were active in the investigated biogas digesters.

## Conclusion

This study revealed that the dominant cellulolytic bacteria in the biogas digesters utilized multiple lignocellulose degradation mechanisms, including using diverse lignocellulases to hydrolyze lignocellulose, which may be the key to maintain high biogas yields from biogas digesters. Besides, some of the lignocellulases may be applied for second generation biofuel production in future.

## Supporting Information

S1 Fig The refinery assembly approach used to assemble GH-containing contigs from metagenomic reads. Targeted reads were retrieved from the biogas metagenome by BlastX of the reference GH protein sequences against the metagenome data, and then subjected to Geneious assembly. The resulted contigs were further extended from their ends by megaBlast to the metagenomic reads or the contigs from 454 Newbler assembly of all metagenomic reads, to obtain the contigs as long as possible. Manual effort was performed at the final step to inspect the assembly quality and get the final list of GH-containing contigs.
(DOCX)

S2 Fig Phylogenetic diversity of the biogas digester metagenomes. (a) Distribution of bacterial 16S rRNA gene sequences was calculated for biogas digesters Z7 and Z8, and compared between PCR amplicons (Z7-PCR and Z8-PCR) and metagenomic reads (Z7-meta and Z8-meta) within each sample. (b) Distribution of archaeal 16S rRNA gene sequences in the tree was calculated for Z7 and Z8, and compared between PCR amplicons and metagenomic reads within each sample. (c) Phylogenetic tree of 16S rRNAs from both PCR amplification and metagenomic sequencing. Sequences were inserted into an ARB reference tree (Download from greengenes database,http://greengenes.lbl.gov/), with phylogenetic grouping retained from the original topology. The number in each branch represent the total number of 16S rRNAs from both PCR amplification and metagenomics sequencing identified in this study.
(DOCX)

S3 Fig The 16S rRNA gene phylogenetic tree was constructed with the lanemaskPH filter using maximum likelihood method. The representative 16S rRNA gene sequences of 16 dominant OTUs were marked with asterisks (*). The scale bar indicates 0.1 nucleotide substitutions per site.
(DOCX)

S4 Fig Taxonomy assignment of NR hits by MEGAN method. (a) Taxonomy assignment of NR hits by MEGAN at different ranks and their distribution in different ranks. 68% of all the Z7 and Z8 metagenomic reads was total assigned at different level. (b) Distribution of dominant phyla in Z7 and Z8 metagenomes analyzed by MEGAN from NR hits (only phyla with ≥0.1% abundance in Z7 and Z8 metagenomes were shown in the figure).
(DOCX)

S5 Fig Gene organizations of the fosmid contigs assembled with sequences of fomid clones. Red, GHs and other lignocellulases; purple, regulatory proteins; Grey, Ribosomal RNA; Dark green, proteins in transport systems; Blue, diverse functions and hypothetical protein without known domains; Black box, unknown sequence in the fosmid contigs. The GH-containing contigs in the fosmid contigs were showed in parallel.
(DOCX)

S6 Fig Phylogenetic tree of six full-length GH5 genes (marked with red) recovered from the biogas digesters and their nearest neighbors was constructed based on maximum likelihood with 100 bootstrap replications. All bootstrap values are displayed in the tree. Sequences of subfamily GH5_4 were marked with pink; Sequences of subfamily GH5_10 were marked with blue; Unclassified GH5 family sequences were marked with black. The accession numbers and origins of the reference GH5 sequences were shown on the tree. The scale bar indicates 0.3 amino acid substitutions per site.
(DOCX)

S1 File Supplementary materials and methods. (DOC)

S2 File All the bacterial OTUs in 16S rRNA gene clone libraries of Z7 and Z8. Including Sheet1-simple information and Sheet2-detail information.
(XLS)

S3 File All the archaeal OTUs in 16S rRNA gene clone libraries of Z7 and Z8. Including Sheet 1-simple information and Sheet 2-detail information.
(XLS)

S1 Table Primers used for verification of GH-containing contigs assembled from the metagenomic short reads by the refined assembly method and screening of fosmid clones harboring these GH-containing or BG-1 16S rRNA gene. (DOCX)

S2 Table Strains and plasmids used in this study. (DOCX)

S3 Table Primers used for cloning and expression of six GH5 family genes in this study. (DOCX)

S4 Table Diversity of microbial communities of Z7 and Z8 based on analysis of 16S rRNA gene clone libraries. (DOCX)

S5 Table Phylogenetic distribution of 16S rRNA genes from clone libraries and metagenomic data. (DOCX)

S6 Table Overview of sequence annotation of metagenomes. (DOCX)

S7 Table Dominant species^1^ identified by MEGAN analysis from both metagenomes. (DOCX)

S8 Table Metagenomic reads encoding glycoside hydrolase genes. (DOCX)

S9 Table Metagenomic reads encoding CBM identified in the Z7 and Z8 metagenomes. (DOCX)

S10 Table Metagenomic reads encoding cellulosomal genes in Z7 and Z8 metagenomes. (DOCX)

S11 Table Numbers of reads or contigs in the GH-containing contigs recovering process with the refinery assembly approach in this study. (DOCX)

S12 Table The 163 GH-containing contigs assembled from the metagenomic data of biogas digesters with the refined assembly approach. (DOCX)

S13 Table The 214 contigs assembled with Newbler software from the metagenomic reads of Z7 and Z8 assigned to BG-1 by PhyloPythiaS analysis. (DOCX)

S14 Table Chemical analysis of slurry and biogas production data of the two biogas digesters. (DOCX)
