# Supplementary material for: Insight into Dominant Cellulolytic Bacteria from Two Biogas Digesters and Their Glycoside Hydrolase Genes
Source: PLoS One. 2015 Jun 12;10(6):e0129921. doi: 10.1371/journal.pone.0129921 (PMC4466528; doi:10.1371/journal.pone.0129921)
Supplement: S1 Fig — Targeted reads were retrieved from the biogas metagenome by BlastX of the reference GH protein sequences against the metagenome data, and then subjected to Geneious assembly. The resulted contigs were further extended from their ends by megaBlast to the metagenomic reads or the contigs from 454 Newbler assembly of all metagenomic reads, to obtain the contigs as long as possible. Manual effort was performed at the final step to inspect the assembly quality and get the final list of GH-containing contigs. (DOCX) [file pone.0129921.s001.docx]

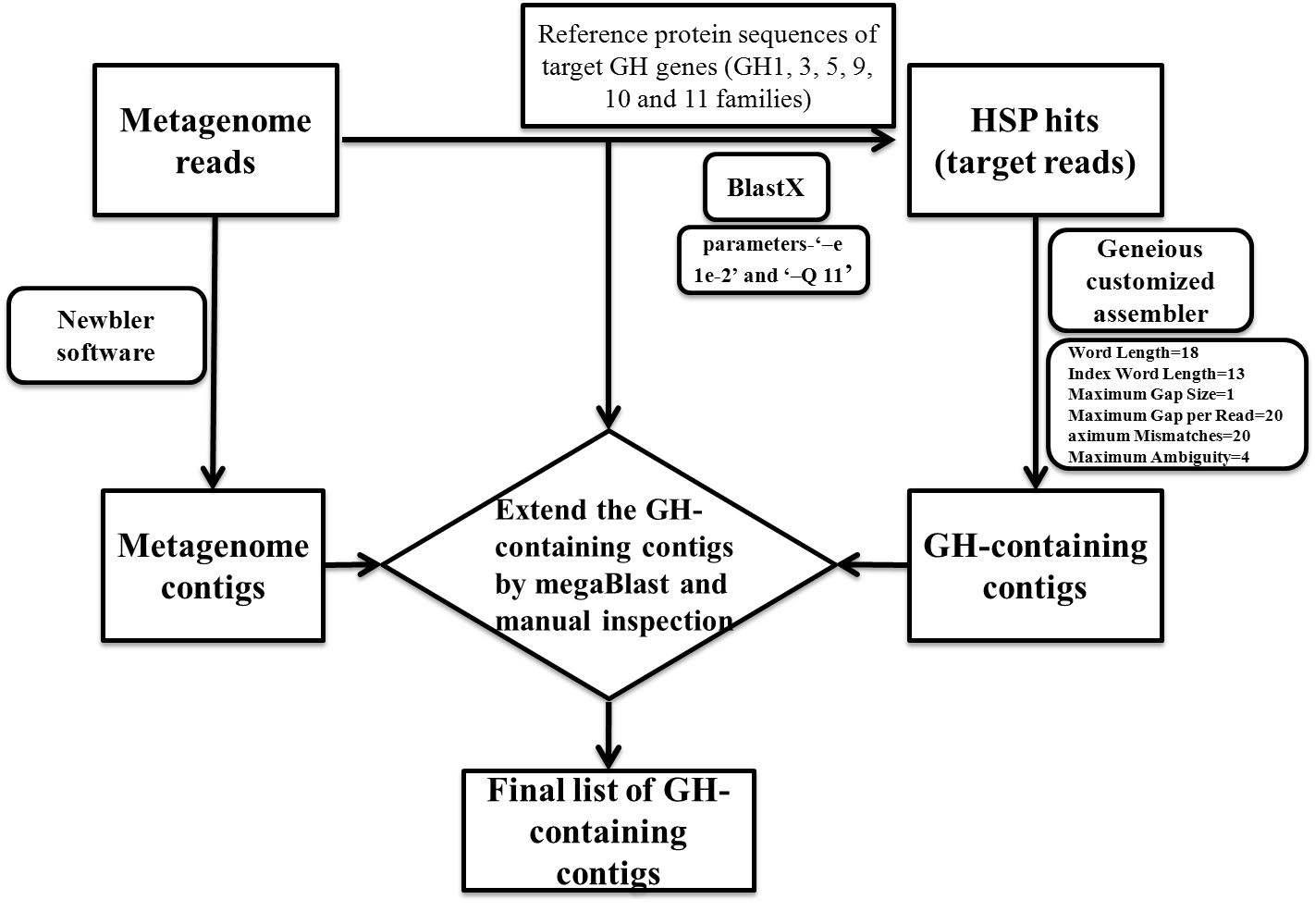


**S1 Fig.** The refinery assembly approach used to assemble GH-containing contigs from metagenomic reads. Targeted reads were retrieved from the biogas metagenome by BlastX of the reference GH protein sequences against the metagenome data, and then subjected to Geneious assembly. The resulted contigs were further extended from their ends by megaBlast to the metagenomic reads or the contigs from 454 Newbler assembly of all metagenomic reads, to obtain the contigs as long as possible. Manual effort was performed at the final step to inspect the assembly quality and get the final list of GH-containing contigs.
