# Supplementary material for: Insight into Dominant Cellulolytic Bacteria from Two Biogas Digesters and Their Glycoside Hydrolase Genes
Source: PLoS One. 2015 Jun 12;10(6):e0129921. doi: 10.1371/journal.pone.0129921 (PMC4466528; doi:10.1371/journal.pone.0129921)
Supplement: S2 Fig — (a) Distribution of bacterial 16S rRNA gene sequences was calculated for biogas digesters Z7 and Z8, and compared between PCR amplicons (Z7-PCR and Z8-PCR) and metagenomic reads (Z7-meta and Z8-meta) within each sample. (b) Distribution of archaeal 16S rRNA gene sequences in the tree was calculated for Z7 and Z8, and compared between PCR amplicons and metagenomic reads within each sample. (c) Phylogenetic tree of 16S rRNAs from both PCR amplification and metagenomic sequencing. Sequences were inserted into an ARB reference tree (Download from greengenes database,http://greengenes.lbl.gov/), with phylogenetic grouping retained from the original topology. The number in each branch represent the total number of 16S rRNAs from both PCR amplification and metagenomics sequencing identified in this study. (DOCX) [file pone.0129921.s002.docx]

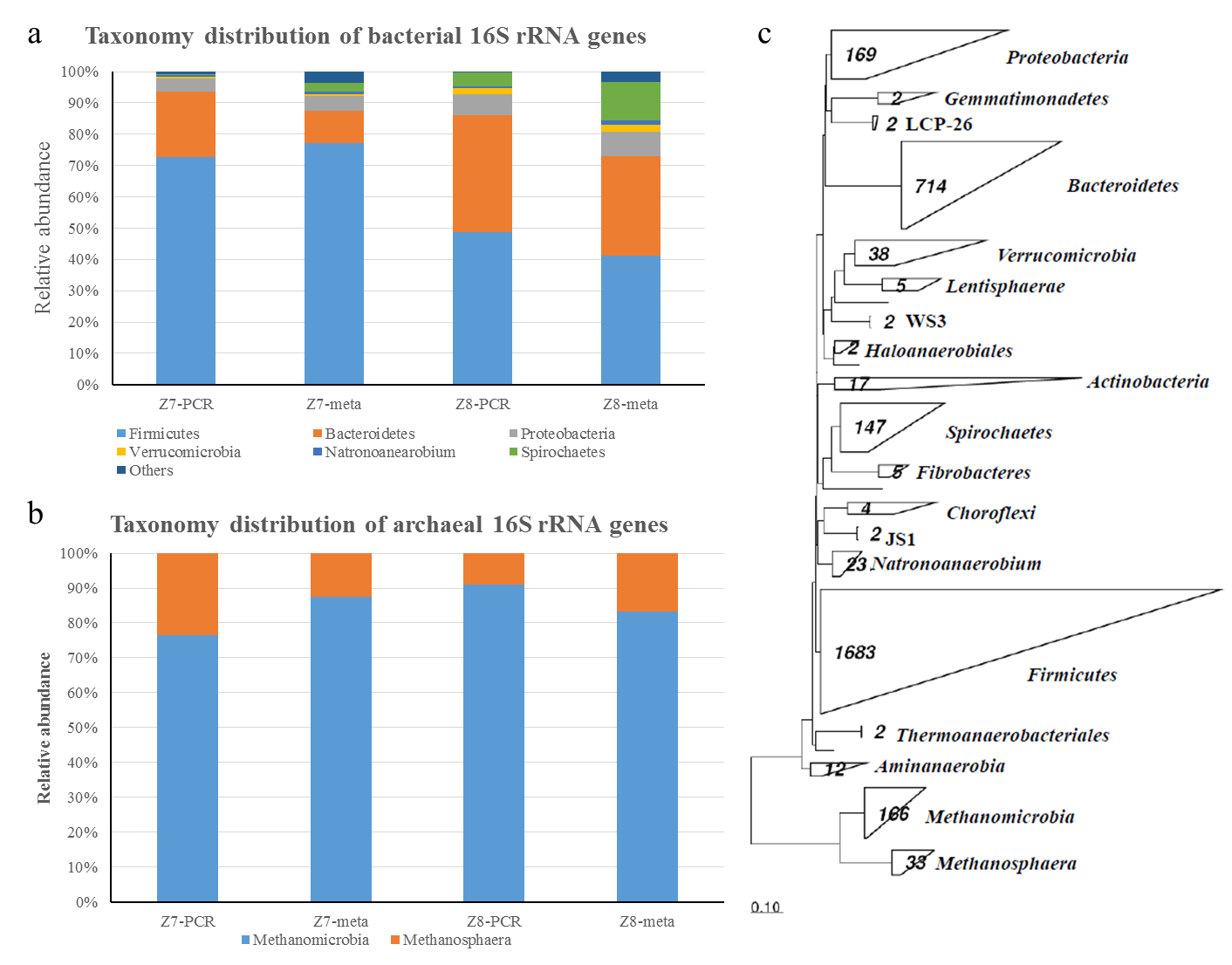


**S2 Fig.** Phylogenetic diversity of the biogas digester metagenomes. (a) Distribution of bacterial 16S rRNA gene sequences was calculated for biogas digesters Z7 and Z8, and compared between PCR amplicons (Z7-PCR and Z8-PCR) and metagenomic reads (Z7-meta and Z8-meta) within each sample. (b) Distribution of archaeal 16S rRNA gene sequences in the tree was calculated for Z7 and Z8, and compared between PCR amplicons and metagenomic reads within each sample. (c) Phylogenetic tree of 16S rRNAs from both PCR amplification and metagenomic sequencing. Sequences were inserted into an ARB reference tree (Download from greengenes database, <http://greengenes.lbl.gov/>), with phylogenetic grouping retained from the original topology. The number in each branch represent the total number of 16S rRNAs from both PCR amplification and metagenomics sequencing identified in this study.
