# Supplementary material for: Insight into Dominant Cellulolytic Bacteria from Two Biogas Digesters and Their Glycoside Hydrolase Genes
Source: PLoS One. 2015 Jun 12;10(6):e0129921. doi: 10.1371/journal.pone.0129921 (PMC4466528; doi:10.1371/journal.pone.0129921)
Supplement: S3 Fig — The representative 16S rRNA gene sequences of 16 dominant OTUs were marked with asterisks (*). The scale bar indicates 0.1 nucleotide substitutions per site. (DOCX) [file pone.0129921.s003.docx]

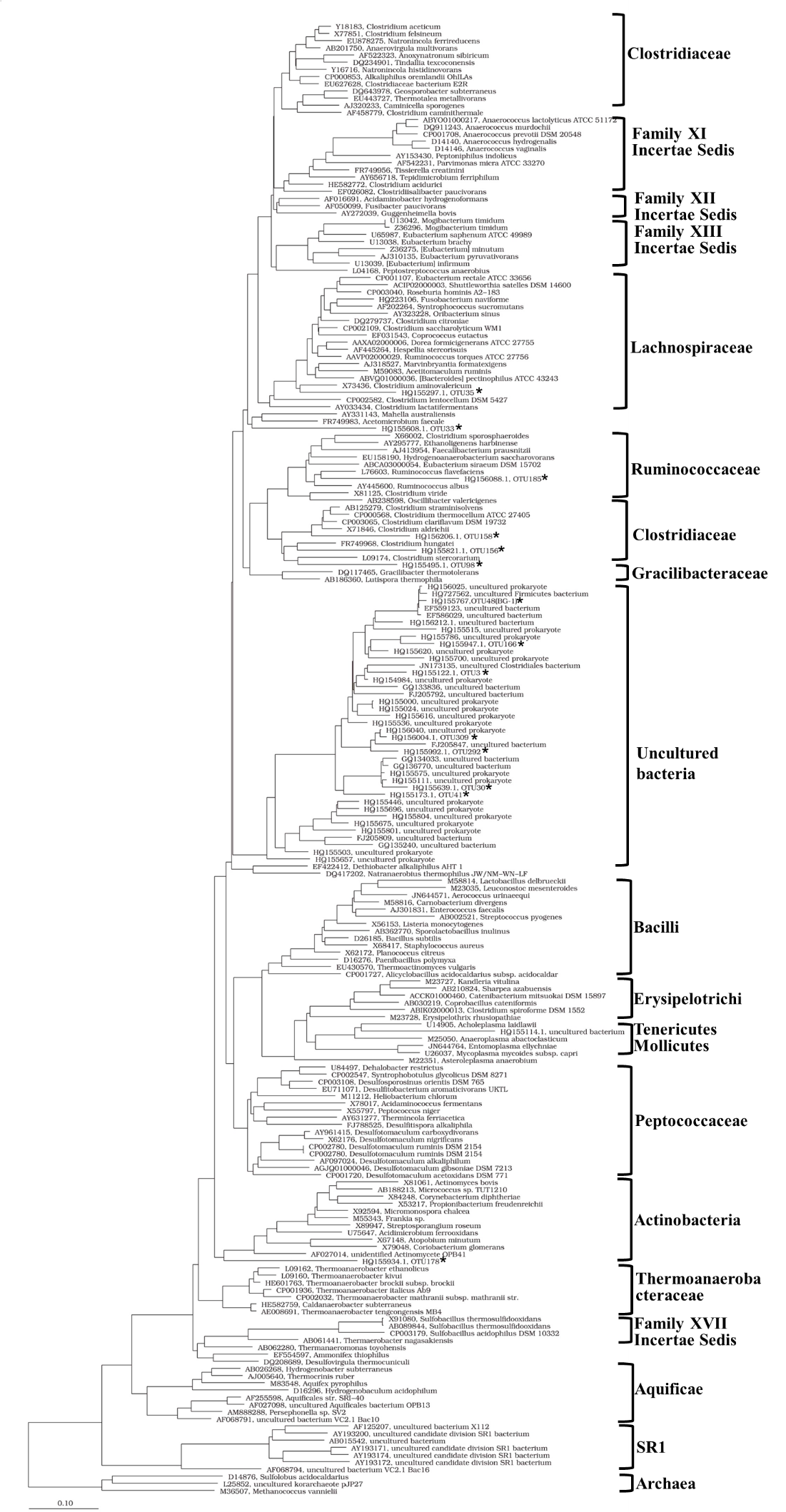


**S3 Fig.** The 16S rRNA gene phylogenetic tree was constructed with the lanemaskPH filter using maximum likelihood method. The representative 16S rRNA gene sequences of 16 dominant OTUs were marked with asterisks (*). The scale bar indicates 0.1 nucleotide substitutions per site.
