# Supplementary material for: Insight into Dominant Cellulolytic Bacteria from Two Biogas Digesters and Their Glycoside Hydrolase Genes
Source: PLoS One. 2015 Jun 12;10(6):e0129921. doi: 10.1371/journal.pone.0129921 (PMC4466528; doi:10.1371/journal.pone.0129921)
Supplement: S4 Fig — (a) Taxonomy assignment of NR hits by MEGAN at different ranks and their distribution in different ranks. 68% of all the Z7 and Z8 metagenomic reads was total assigned at different level. (b) Distribution of dominant phyla in Z7 and Z8 metagenomes analyzed by MEGAN from NR hits (only phyla with ≥0.1% abundance in Z7 and Z8 metagenomes were shown in the figure). (DOCX) [file pone.0129921.s004.docx]

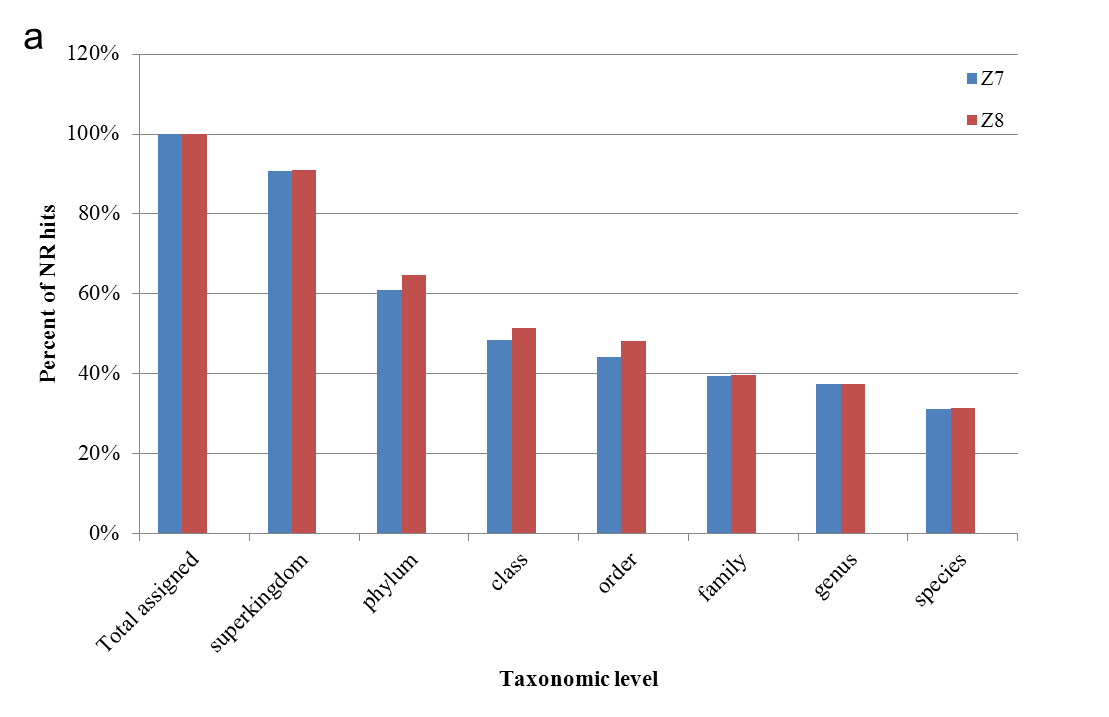


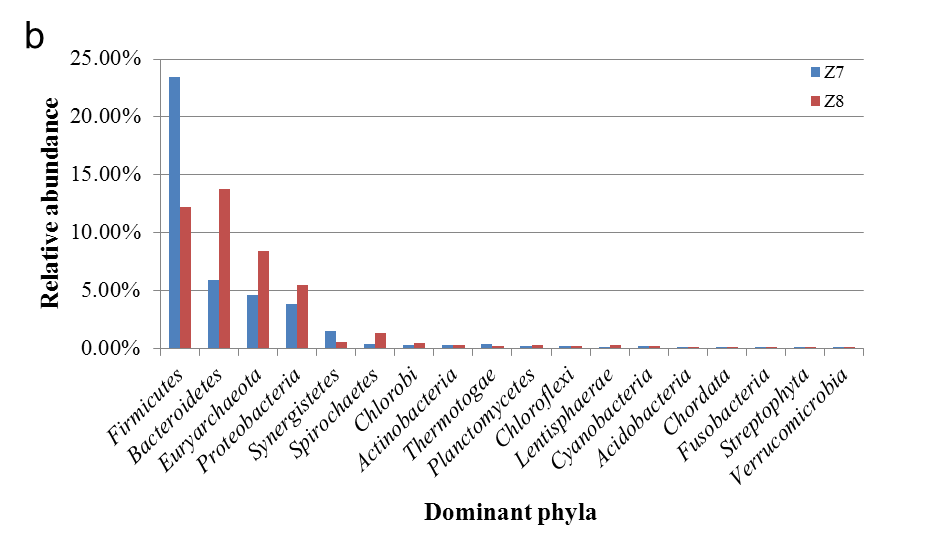


**S4 Fig.** Taxonomy assignment of NR hits by MEGAN method. (a) Taxonomy assignment of NR hits by MEGAN at different ranks and their distribution in different ranks. 68% of all the Z7 and Z8 metagenomic reads was totally assigned at different level. (b) Distribution of dominant phyla in Z7 and Z8 metagenomes analyzed by MEGAN from NR hits (only phyla with ≥0.1% abundance in Z7 and Z8 metagenomes were shown in the figure).
