# Supplementary material for: Insight into Dominant Cellulolytic Bacteria from Two Biogas Digesters and Their Glycoside Hydrolase Genes
Source: PLoS One. 2015 Jun 12;10(6):e0129921. doi: 10.1371/journal.pone.0129921 (PMC4466528; doi:10.1371/journal.pone.0129921)
Supplement: S5 Fig — Red, GHs and other lignocellulases; purple, regulatory proteins; Grey, Ribosomal RNA; Dark green, proteins in transport systems; Blue, diverse functions and hypothetical protein without known domains; Black box, unknown sequence in the fosmid contigs. The GH-containing contigs in the fosmid contigs were showed in parallel. (DOCX) [file pone.0129921.s005.docx]

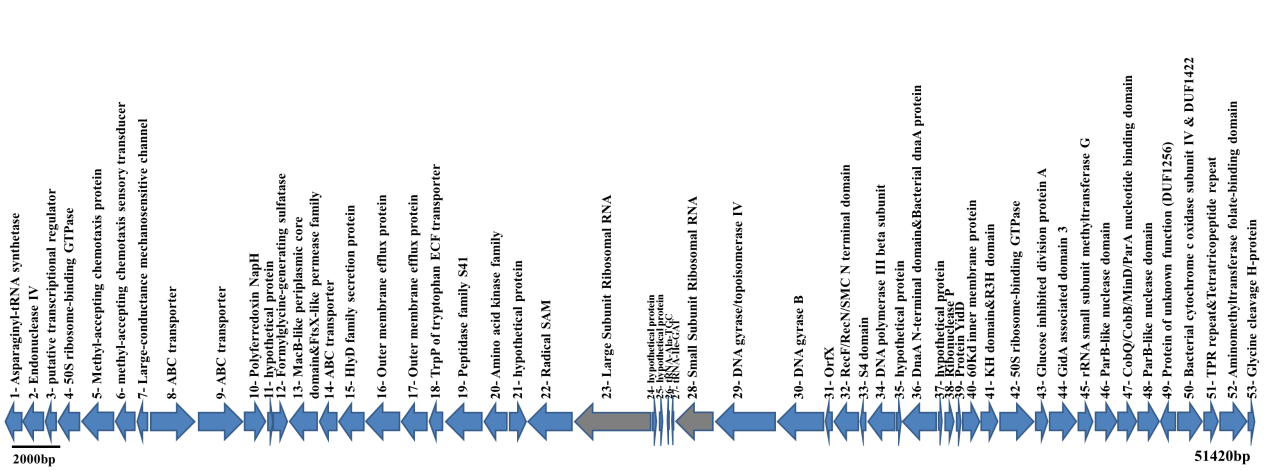


Contig FC3 (assembly of sequences of Fosmid81E17 and Fosmid83P13)


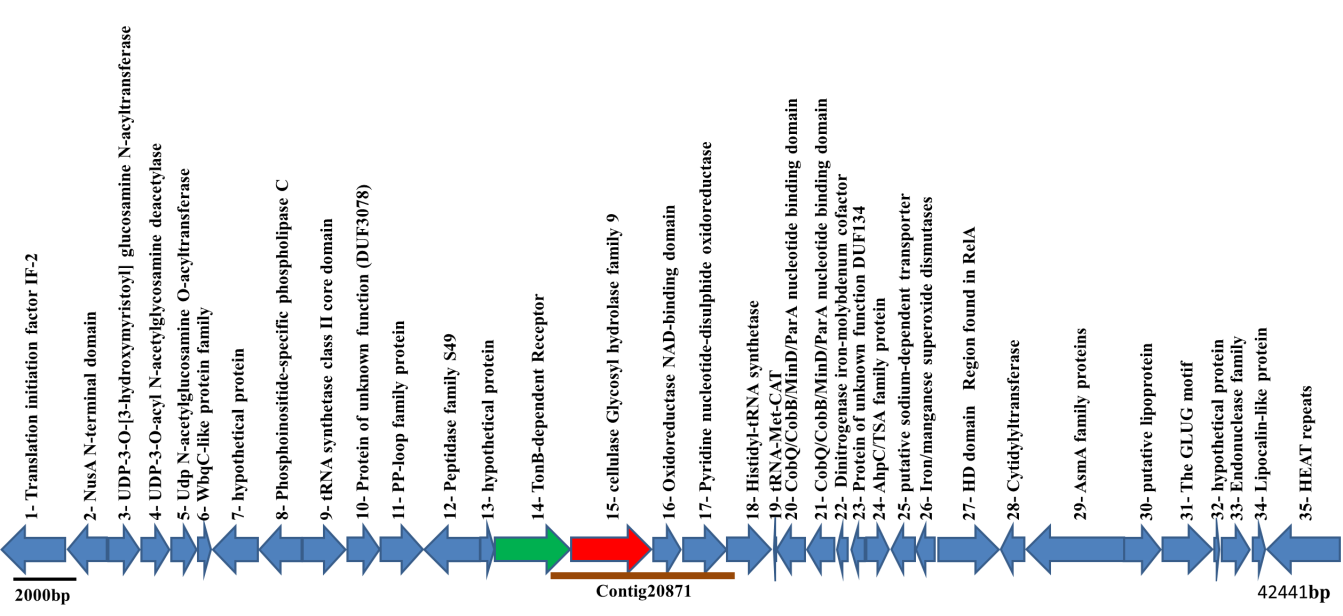


Contig FC4 (assembly of sequences of Fosmid86J4)


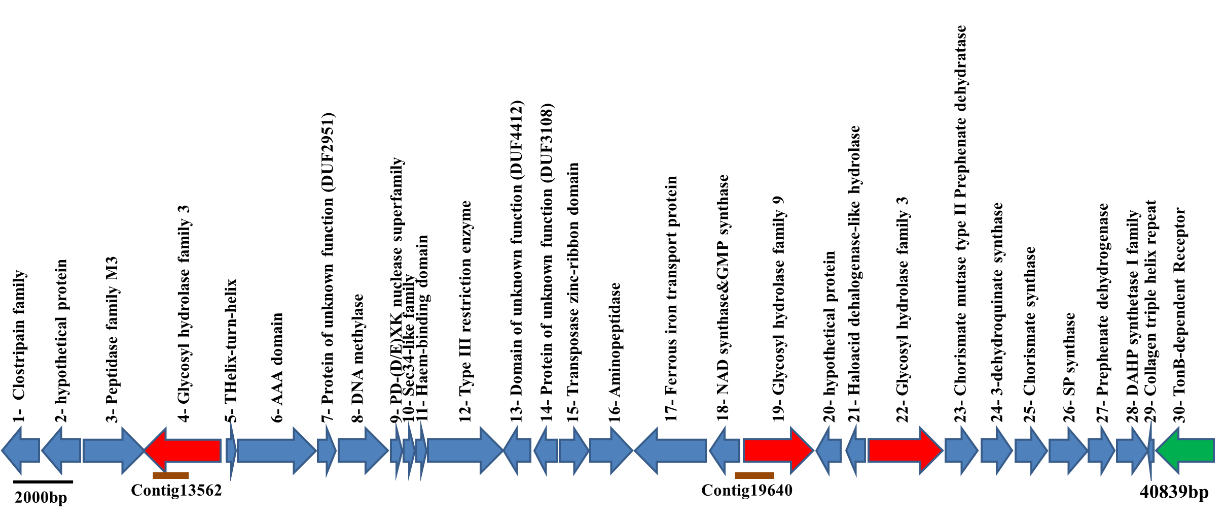


Contig FC5 (assembly of sequences of Fosmid93D17)


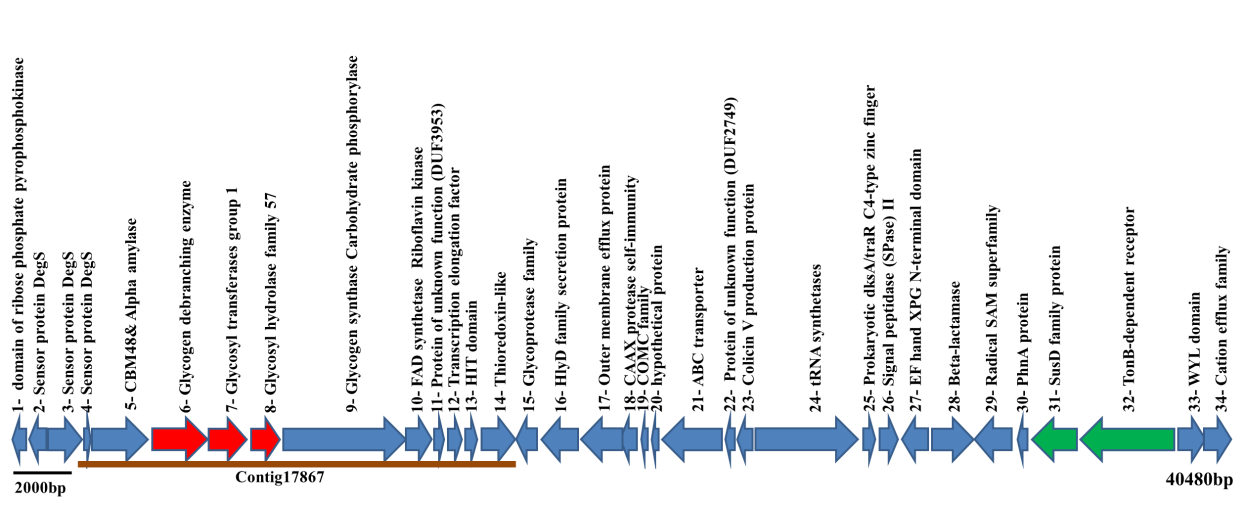


Contig FC6 (assembly of sequences of Fosmid251K21)


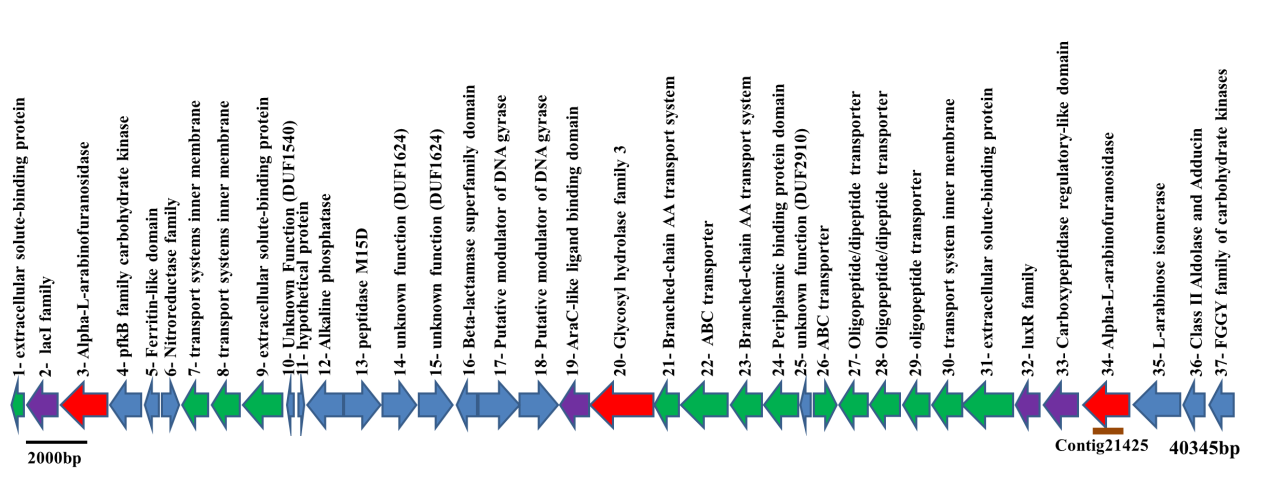


Contig FC7 (assembly of sequences of Fosmid276P23)


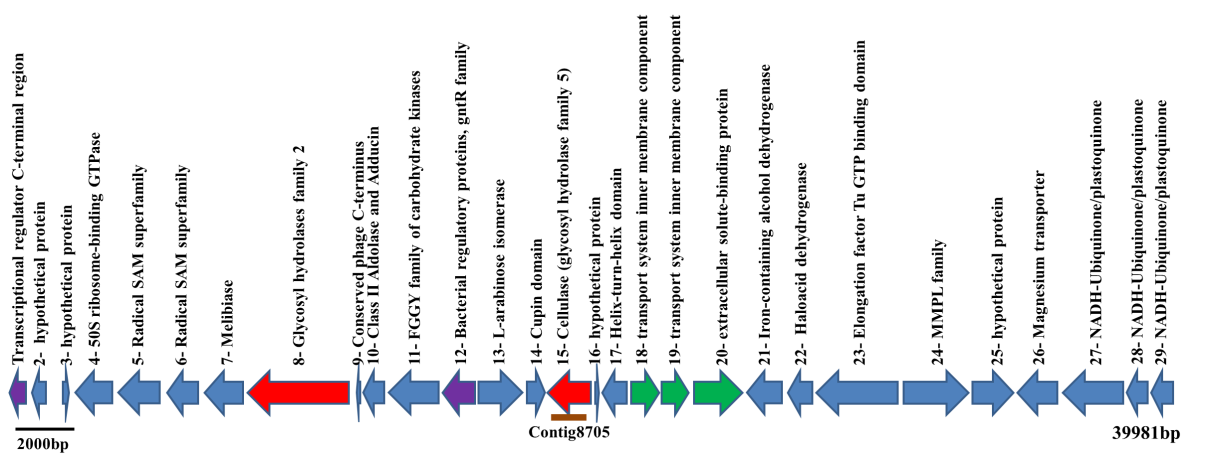


Contig FC8 (assembly of sequences of Fosmid82I22)


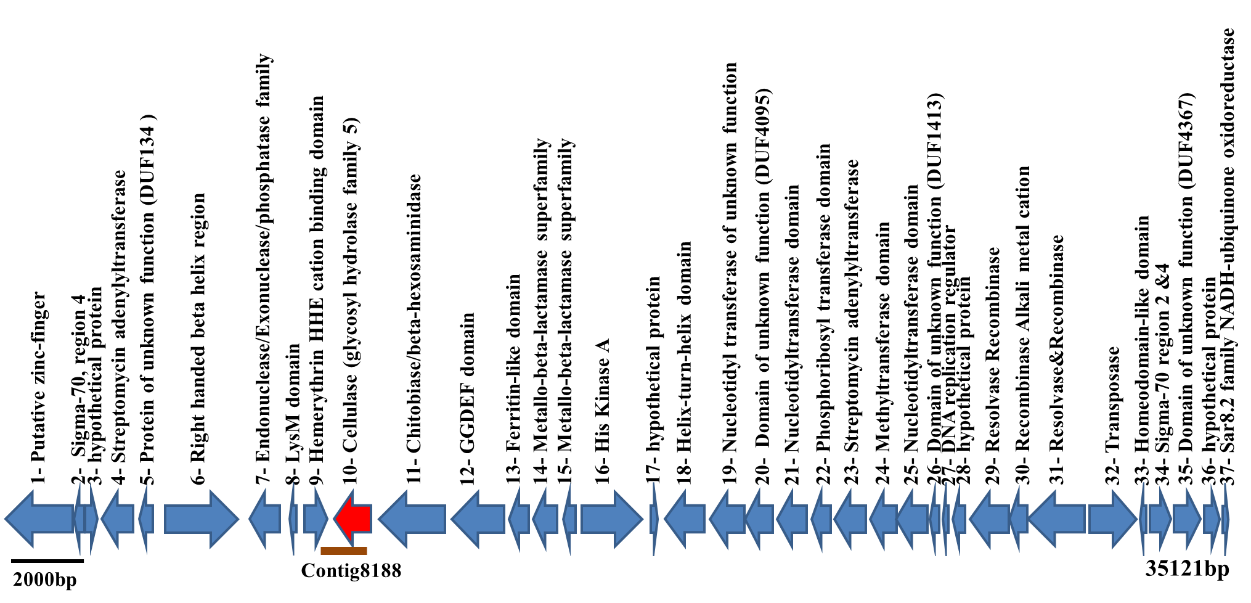


Contig FC9 (assembly of sequences of Fosmid88I5)


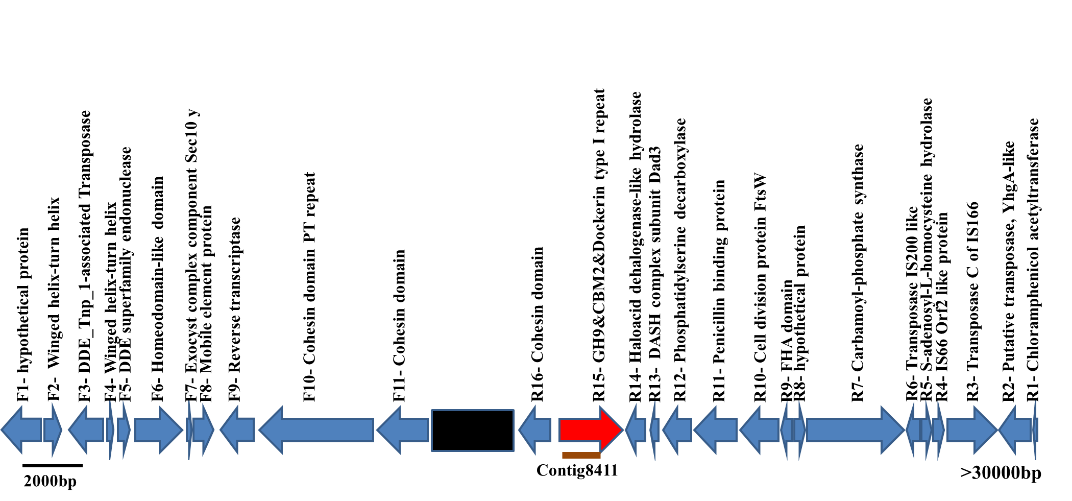


Contig FC10F and FC10R (assembly of sequences of Fosmid255O19)

**S5 Fig.** Gene organizations of the fosmid contigs assembled with sequences of fomid clones. Red, GHs and other lignocellulases; purple, regulatory proteins; Grey, Ribosomal RNA; Dark green, proteins in transport systems; Blue, diverse functions and hypothetical protein without known domains; Black box, unknown sequence in the fosmid contigs. The GH-containing contigs in the fosmid contigs were showed in parallel.
