# Supplementary material for: Insight into Dominant Cellulolytic Bacteria from Two Biogas Digesters and Their Glycoside Hydrolase Genes
Source: PLoS One. 2015 Jun 12;10(6):e0129921. doi: 10.1371/journal.pone.0129921 (PMC4466528; doi:10.1371/journal.pone.0129921)
Supplement: S6 Fig — All bootstrap values are displayed in the tree. Sequences of subfamily GH5_4 were marked with pink; Sequences of subfamily GH5_10 were marked with blue; Unclassified GH5 family sequences were marked with black. The accession numbers and origins of the reference GH5 sequences were shown on the tree. The scale bar indicates 0.3 amino acid substitutions per site. (DOCX) [file pone.0129921.s006.docx]

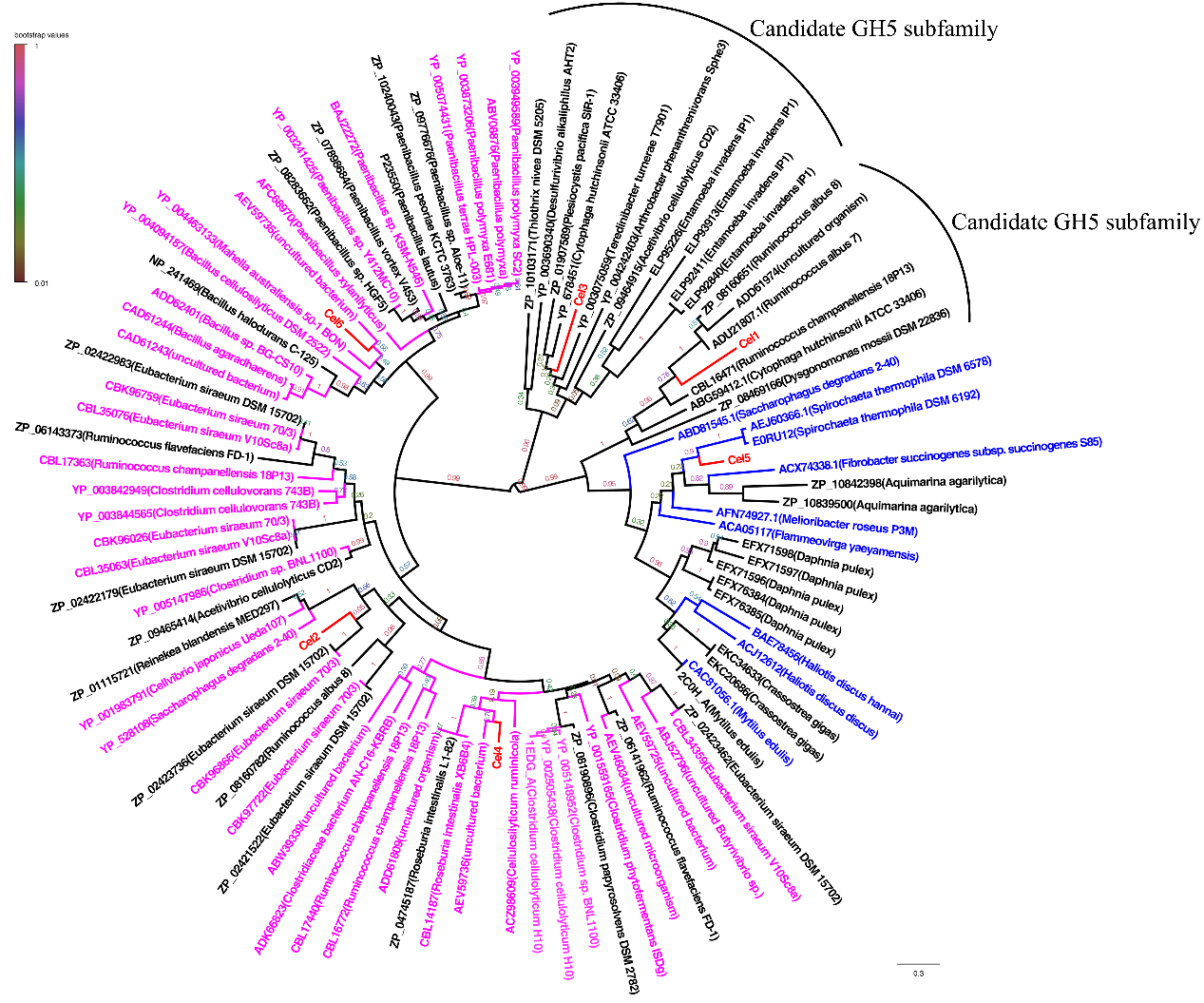


**S6 Fig.** Phylogenetic tree of six full-length GH5 genes (marked with red) recovered from the biogas digesters and their nearest neighbors was constructed based on maximum likelihood with 100 bootstrap replications. All bootstrap values are displayed in the tree. Sequences of subfamily GH5_4 were marked with pink; Sequences of subfamily GH5_10 were marked with blue; Unclassified GH5 family sequences were marked with black. The accession numbers and origins of the reference GH5 sequences were shown on the tree. The scale bar indicates 0.3 amino acid substitutions per site.
