# Supplementary material for: Insight into Dominant Cellulolytic Bacteria from Two Biogas Digesters and Their Glycoside Hydrolase Genes
Source: PLoS One. 2015 Jun 12;10(6):e0129921. doi: 10.1371/journal.pone.0129921 (PMC4466528; doi:10.1371/journal.pone.0129921)
Supplement: S1 File — (DOC) [file pone.0129921.s007.doc]

**Supplementary Materials and Methods**

**Chemical analysis**

NH3-N concentration of the slurry at the end of the fermentation was measured on a PC spectro Photometer (Lovibond, Dortmund, Germany) by utilization of the method 506 NH3-N concentration (Tintometer GmbH, Dortmund, Germany) for NH3-N determination in the 0-50 mg/l range (Samples was diluted to this range). Biogas was collected to measure biogas volume at room temperature. Biogas composition was analyzed with a gas chromatograph GC7900 (Techcomp, China) equipped with a thermal conductivity detector. A 5-m, stainless, transformer oil analysis column was packed with acidic ethyl acetate (AE) and nitrogen was used as the carrier gas at a flow rate of 16 mL/min. The temperature of the injector, column, and detector were kept at 100, 100, and 130℃, respectively. C and N contents in samples were determined by an elemental analyzer (Perkin Elmer Series II CHNS/O Analyzer 2400).

**16S rRNA gene based analysis of microbial composition in two biogas digesters Z7 and Z8**

DNA extracted from samples Z7 and Z8 were subjected to constructed PCR 16S rRNA gene clone library. Primer pairs 27f (5′-AGAGTTTGATCMTGGCTCAG-3′)/1492r (5′- GGTTACCTTGTTACGACTT-3′) and Arch-571F (5’-GCYTAAAGSRICCGTAGC-3’)/ Univ-1406R (5’-ACGGGCGGTGWGTRCAA-3’) were used to amplify 16S rRNA gene of bacterial and archaeal domain respectively. The PCR mix contained 2.5 μL of 10×Ex Taq buffer, 2.5 μL of 2 mM dNTPs, 10 pmol of each primer, 0.5 U of Ex Taq DNA polymerase (Takara Biotechnology (Dalian) Co., Ltd.), and 5 ng of template DNA in a total volume of 25 μL. The following cycling parameters were used for bacterial 16S rRNA gene amplification: at 95°C for 1 min and 30 s and then cycled 18 times through the following temperature profile: 95°C for 30 s, annealing temperature (Ta) for 30 s, and 72°C for 4 min. The Ta was 60°C for the first 6 cycles, 55°C for the next 6 cycles, and 50°C for the last 6 cycles. As for amplification of archaeal 16S rRNA gene, 25 cycles were used and Ta was 60°C for the first 5 cycles, 55°C for the next 5 cycles, and 50°C for the last 15 cycles. In order to reduce heteroduplexes in PCR product, reconditioning PCR was carried out (1). Clone libraries constructed from PCR amplicons of 16S rRNA genes from Z7 and Z8 biogas digesters were chosen randomly and sequenced. After assembled with Geneious software, all sequences were aligned using the NAST aligner from the Greengenes website (<http://greengenes.lbl.gov/>) (2, 3). All possible chimeras were detected with Bellerophon (version 3) hosted at Greengenes and then excluded from subsequent analysis (4). The resulting aligned sequences were imported into ARB for phylogenetic analysis (5). Filters were constructed to exclude ambiguously aligned regions, and the remaining aligned regions were subjected to distance matrix calculation, and a correction model was selected automatically in ARB. The calculated distance matrixes for both bacterial and archaeal sequences were used to calculate operational taxonomic units (OTUs) (similarity cutoff value ≥ 99%) using DOTUR software (6). The representative sequences of each OTU were submited to RDP project database for phylum classification. STAMP (7) was used to identify significant different OTUs between Z7 and Z8. ‘Two samples’ method from STAMP software was used for the analysis (two-sided permutation test, 0.95 CI of ‘Asymptotic-CC’, Storey FDR corrected q-values).

16S rRNA gene sequences in the metagenomic data were predicted using BLASTN against the RDPII database (version 9.58). For each 16S rRNA gene match, reads with an e-value of ≤ 10^-5^, a match length of ≥ 65 nt, and a similarity ≥ 80% were retrieved for further analysis (8).

To compare the phylogenetic profiles of the 16S rRNA genes between PCR amplicons and metagenome predictions, sequences from both datasets were aligned with NAST at Greengenes and imported into ARB software, and then inserted into the reference tree in the database (all in the tree list) with the lanemaskPH filter using the parsimony insertion algorithm. From the resulting phylogenetic tree, groups of phyla were identified and the abundance of included sequences was calculated for PCR amplicons and for the metagenome, respectively.

**MEGAN analysis of microbial compositions in the metagenomes**

NCBI taxonomy was assigned to each read using MEGAN (9) with the following parameters. In brief, MEGAN uses a lowest common ancestor (LCA) algorithm to assign taxonomy to reads. For each read, BLASTX may return top-hits (filtered by MEGAN based on alignment bit-score, default parameters) of different taxonomy. Therefore, to assign a consistent taxonomy, the lowest common ancestor of these top-hits is identified as the most recent common taxonomic ranks to all these hits, starting from root. In this way, the most recent common taxonomy is used to assign the read matching these hits. The *min-score* filter was set to ≥ 35.0, given the short length of analyzed reads, to discard low-score matches that may be randomly aligned. The *top-percentage* filter was set to 90% to include the high-score matches, including scores no less than 90% of the highest score. The *win-score* and *min-support* filters were unspecified. Reads were binned into different taxa, from superkingdom to species. These taxonomic bins were used to compare the microbial composition between Z7 and Z8 at different taxonomic levels.

**Comparative analysis**

Enrichment or depletion of function categories or taxonomic groups between Z7 and Z8 was tested statistically with XIPE (version 0.01), a software using bootstrap technique to compare difference between samples (10). The following parameters were set for resampling: subsample size of 10,000, repetitions of 10,000 and three runs of different confidence levels (95%, 98% and 99%). To further establish a confidence level for the calculated difference, the same procedure was performed to the same category with sampling randomly from mixed Z7 and Z8 datasets. And a difference value outside the specified confidence interval was considered to be significant.

**Analysis of Glycoside hydrolase (GH), carbohydrate-binding modules (CBM) and cellulosomal genes in the biogas digesters**

To identify GHs and CBMs in the metagenomes, CAZy (carbohydrate-active enzymes, http://www.cazy.org) was used as the reference classification system of GH and CBM families (11). For those GH or CBM families have associated Pfam HMM models, the corresponding fragment models were downloaded from the Pfam website (<http://pfam.sanger.ac.uk/)> for the search of GHs and CBMs in local HMM model and local sequence with multiple domains (12). Translated protein sequence for each read was determined by BLASTX against NCBI-NR database. In the case of multiple coding regions or frame-shift for one single read indicated by BLASTX HSPs of the best hit, all the matched segments were retrieved to make up a protein database of the metagenomes. Hmmsearch in the hmmer package was then used to search the compiled protein database for GHs and CBMs using the downloaded Pfam HMMs and default e-value threshold (13). In the case of GHs and CBMs with no Pfam HMM available, representative sequences were determined for them (14). Hits with e-value ≤ 10^-6^ were retrieved from BLASTX of the metagenome against these selected sequences. Results of both analyses were referred to CAZy database to incorporate enzyme activity information. For those GHs and CBMs with multiple activities for one single record, its matched KOs from the KEGG database were taken into account to select the most possible activity information. Protein sequences of typical cellulosomal genes were downloaded from NCBI to establish a BLAST database (15). The whole metagenome was screened for possible cellulosomal gene coding reads using BLASTX with the E-value cutoff ≤ 1e-5. Matched reads were then manually inspected for alignment regions of HSPs to select the significant hits to annotation such cellulosomal genes.

**Verification of 50 GH-containing contigs recovered by the refinery assembly method**

Verification of GH-containing contigs recovered by the refinery assembly method was carried out by PCR using Z7 metagenomic DNA as template. Primers used for PCR verification were listed in Supplementary Table S1. The PCR was performed in a 25-μL reaction mixture containing 0.75 U of rTaq DNA polymerase (Takara Biotechnology (Dalian) Co., Ltd.), 2.5 μL of 10 × rTaq buffer, 2.5 μL of 2 mM dNTPs, 0.4 μM of each primer, and 5 ng of sample DNA. The PCR was performed 94ºC for 5 mins, followed by 40 cycles of denaturation (30 seconds, 94ºC), annealing (30 seconds, 52ºC) and extension (1kb PCR fragment per minute, 72ºC), finally 72ºC for 10 mins. If there were no predicted PCR products, the annealing temperature was refined for more try. The amplified PCR products were checked by electrophoresis on agarose gel and then sequenced.

**Screening, sequencing and annotation of positive fosmid clones harboring GH-containing contigs from Z7 fosmid clone library**

When screening of positive fosmid clones harboring GH-containing contigs from Z7 fosmid clone library, the PCR reaction mixture and conditions were the same with the PCR protocol described above except that 2 μL of overnight culture of fosmid clones were used as templates. To screen positive clones efficiently, fosmid clones from each 384-well plate were pooled and extracted for fosmid DNA. The fosmid DNA from each plate was tested for screening primers in order to locate positive plates. Then overnight culture collected from each column or row of the positive plates were mixed together and used for PCR amplification to find positive fosmid clones. Then eleven selected positive fosmid clones harboring GH genes and one fosmid clone harboring BG-1 16S rRNA gene were sequenced by 454 pyrosequencing as described before (16). For accurate assignments obtained by the PhyloPythiaS analysis using sample-specific model need about ≥ 50kb reference sequence harboring BG-1 16S rRNA gene, another fosmid clone harboring BG-1 16S rRNA gene was sequenced using Sanger method. All the sequencing datas were assembled into 11 fosmid contigs with Newbler 2.6 software and contig of FC3 which harboring BG-1 16S rRNA gene was used as the sample specific data in PhyloPythiaS analysis (17). After assigning of BG-1 to a new order in *Clostridia* by 16S rRNA gene analysis, a Newick tree was constructed according to NCBI taxonomic identifiers and used as the reference tree in PhyloPythiaS analysis. In order to obtain more BG-1 genome information, the fosmid contigs and the contigs assembled from the metagenomic data (not including the GH-containing contigs recovered by the refinery assembly approach) ≥ 1 kb were used as the prediction datas respectively. The contigs and fosmid contigs assigned to BG-1 were listed in the Table 3 and Supplementary Table S15.

**Calculation of GH-containing contigs and fosmid contigs depth in the biogas digester metagenomic data**

The depth of contigs assembled with Newbler software, GH-containing contigs and fosmid contigs was calculated as follows: based on the assembly results, the number of metagenomic reads which used for assembly of each contigs or which binned to the fosmid contigs (with ≥ 98% similarity) was counted and multiplied by the median read length (253bp and 254bp for Z7 and Z8 metagenomic reads, respectively), and then divided by the length of contigs or fosmid contigs (18). The calculated formula for each contig was: Contig depth = (Z7^Num^* 253+ Z8^Num^* 254)/contigs length; Z7 ^Num^ and Z8^Num^ represent the number of metagenomic reads of Z7 and Z8 used to assembly the corressponding contigs or binned to the fosmid contigs, 253 and 254 represent for the medium length of Z7 and Z8 metagenomic reads.

**Phylogenetic analysis of the dominant *Firmicutes* OTUs with their 16S rRNA gene sequences**

For phylogenetic analysis of BG-1 and other 14 dominant *Firmicutes* OTUs in the biogas digesters, representative 16S rRNA gene sequences of these OTUs were aligned with their nearest sequences of uncultured bacteria and representative sequences of isolates retrieved from silva database with SINA online (19, 20). The output of the alignments were imported into ARB software, and used to create phylogenetic tree with the lanemaskPH filter using maximum likelihood method (21).

**Phylogenetic analysis of six full-length GH5 family genes**

Six full-length GH5 genes named Cel1 to Cel6 were recovered from GH-containing contigs or fosmid contigs of the fosmid clones, which showed similarities with their nearest neighbors from 49% to 66% (Table 4). Cel1 is in the contig of FC9 (KJ797025; The encoding region of Cel1 starts from 10848bp to 9832bp); Cel2 is in the contig of Contig8330 (KJ797183; The encoding region of Cel2 starts from 2948 to 1179bp); Cel3 is in the contig of Contig8557 (KJ797186; The encoding region of Cel3 starts from 2027bp to 501bp); Cel4 is in the contig of FC8 (KJ797024; The encoding region of Cel4 starts from 20983bp to 19521bp); Cel5 is in the contig of FC1 (KJ797017, ; The encoding region of Cel5 starts from 53398bp to 54522bp); Cel6 is in the contig of FC1 (KJ797017; The encoding region of Cel6 starts from 54553bp to 56238bp). The accession number of Cel5 and Cel6 were also HQ706354.1 and HQ706345.1

For phylogenetic analysis of the six GH5 genes, the amino acid sequences of these six GH5 family genes and their nearest protein sequences retrieved from NCBI database were aligned with MAFTT method (22). The output of MAFFT result was edited by Jalview 2.7 and used to create phylogenetic tree by MEGA 5.10 using maximum likelihood method with bootstrap values of 100 replicates (23-25). When Cel1 to Cel6 clustered with reference sequences of GH5 subfamilies, they could be assigned to the corresponding GH5 subfamilies. For the definition of subfamilies was phylogenetic clade with ≥ 5 GH5 genes from different organisms (26), two clades in our tree were defined as GH5 subfamily candidates. Besides, SignalP 4.1 server was used to predict the presence of signal peptide in these six GH5 family genes using default parameters except that gram-positive bacteria was chosen as the organism group (27).

**Expression of six GH5 family genes in *Escherichia coli* and enzymatic assays of these six GH5 proteins**

*E. coli* EPI300-T1^R^ was used as the cloning host (Epicentre, San Diego, CA, USA), and *E. coli* BL21 was the expression host (Novagen, Gibbstown, NJ, USA). The expressed plasmid pET-28a was used for six GH5 genes expression in *E.coli* BL21 (Table S2). The six GH5 genes were amplified from Z7 DNA and gel-purified (Table S3). Then the purified DNA was digested with *EcoR* I and *Xho* I or *Sal* I and *Xho* I, ligated into pET-28a vector digested with the same enzymes, and resulting into six new plamids (Supplementary Table S2). The six plasmids, verified by sequencing, were separately transformed into *E. coli* BL21. Finally, the expressed His-tag-containing GH5 proteins were purified using nickel nitrilotriacetic acid (Ni-NTA) agarose resin according to the manufacturer’s instructions (Qiagen, Valencia, CA, USA).

Purified GH5 proteins were used to test for activities on substrates including carboxymethyl-cellulose (CMC) (Sigma, St. Louis, MO, USA), locust bean gum (Sigma, St. Louis, MO, USA), xylan (beechwood) (Sigma, St. Louis, MO, USA) and p-nitrophenyl-D-cellobioside ( pNPC, Sigma, St. Louis, MO, USA) .

Endoglucanase activity of the purified enzymes was assayed by a procedure for measure of reducing sugar named dinitrosalicylic acid (DNS) method (28). The enzymatic reaction was performed by mixing of 50 μL properly diluted enzyme solution with 50 μL 2% CMC dissolve in pH7.4 Na_2_HPO_4_-NaH_2_PO_4_ buffer at 50℃ for 30 minutes. The reaction was stopped by adding 100 μL DNS solution, followed by incubating it at 95℃ for 10 min and cooled by placing the tubes on ice. Photometric assays were read immediately at OD540 using a Varioskan Flash microplate reader (ThermoScientific, Rockford, IL, USA). The activities of mannanase and xylanase were measured with the same method as endoglucanase activity except that 2% CMC was replaced with 2% locust bean gum or 2% xylan and the reaction time for these two activity assays was reduced to 10 minutes.

For test of exocellulase acitivity，the enzymatic reaction mixture contained 20 μL of appropriately diluted enzyme sample and 90 μL 4mM pNPC dissolved in 0.1 M Na_2_HPO_4_-NaH_2_PO_4_ buffer (pH 7.4). After the mixture was incubated at 50℃ for 30 minutes, 100 μl of the mixture were added to 100 μl 2% Na_2_CO_3_ and OD_400_ was read immediately using a Varioskan Flash microplate reader.

One unit of each enzyme activity was defined as the number of μmol of reducing sugar liberated min^-1^ mg^-1^ protein and reported as U/mg protein.

**References**

1. Thompson JR, Marcelino LA, Polz MF. Heteroduplexes in mixed-template amplifications: formation, consequence and elimination by ‘reconditioning PCR’. Nucleic Acids Research. 2002;30(9):2083-8.

2. DeSantis T, Hugenholtz P, Keller K, Brodie E, Larsen N, Piceno Y, et al. NAST: a multiple sequence alignment server for comparative analysis of 16S rRNA genes. Nucleic Acids Research. 2006;34(suppl 2):W394-W9.

3. DeSantis TZ, Hugenholtz P, Larsen N, Rojas M, Brodie EL, Keller K, et al. Greengenes, a chimera-checked 16S rRNA gene database and workbench compatible with ARB. Applied and Environmental Microbiology. 2006;72(7):5069-72.

4. Huber T, Faulkner G, Hugenholtz P. Bellerophon: a program to detect chimeric sequences in multiple sequence alignments. Bioinformatics. 2004;20(14):2317-9.

5. Ludwig W, Strunk O, Westram R, Richter L, Meier H, Buchner A, et al. ARB: a software environment for sequence data. Nucleic acids research. 2004;32(4):1363-71.

6. Schloss PD, Handelsman J. Introducing DOTUR, a computer program for defining operational taxonomic units and estimating species richness. Applied and environmental microbiology. 2005;71(3):1501-6.

7. Parks DH, Tyson GW, Hugenholtz P, Beiko RG. STAMP: statistical analysis of taxonomic and functional profiles. Bioinformatics. 2014;30(21):3123-4.

8. Mou X, Sun S, Edwards RA, Hodson RE, Moran MA. Bacterial carbon processing by generalist species in the coastal ocean. Nature. 2008;451(7179):708-11.

9. Huson DH, Auch AF, Qi J, Schuster SC. MEGAN analysis of metagenomic data. Genome Research. 2007;17(3):377-86.

10. Rodriguez-Brito B, Rohwer F, Edwards RA. An application of statistics to comparative metagenomics. BMC bioinformatics. 2006;7(1):162.

11. Cantarel BL, Coutinho PM, Rancurel C, Bernard T, Lombard V, Henrissat B. The Carbohydrate-Active EnZymes database (CAZy): an expert resource for glycogenomics. Nucleic Acids Research. 2009;37(suppl 1):D233-D8.

12. Finn RD, Mistry J, Schuster-Böckler B, Griffiths-Jones S, Hollich V, Lassmann T, et al. Pfam: clans, web tools and services. Nucleic acids research. 2006;34(suppl 1):D247-D51.

13. Eddy SR. Profile hidden Markov models. Bioinformatics. 1998;14(9):755-63.

14. Warnecke F, Luginbühl P, Ivanova N, Ghassemian M, Richardson TH, Stege JT, et al. Metagenomic and functional analysis of hindgut microbiota of a wood-feeding higher termite. Nature. 2007;450(7169):560-5.

15. Schlüter A, Bekel T, Diaz NN, Dondrup M, Eichenlaub R, Gartemann K-H, et al. The metagenome of a biogas-producing microbial community of a production-scale biogas plant fermenter analysed by the 454-pyrosequencing technology. Journal of Biotechnology. 2008;136(1):77-90.

16. Yan X, Geng A, Zhang J, Wei Y, Zhang L, Qian C, et al. Discovery of (hemi-) cellulase genes in a metagenomic library from a biogas digester using 454 pyrosequencing. Applied Microbiology and Biotechnology. 2013;97(18):8173-82.

17. Patil KR, Roune L, McHardy AC. The PhyloPythiaS web server for taxonomic assignment of metagenome sequences. PLoS ONE. 2012;7(6):e38581.

18. Allgaier M, Reddy A, Park JI, Ivanova N, D'haeseleer P, Lowry S, et al. Targeted discovery of glycoside hydrolases from a switchgrass-adapted compost community. PLoS ONE. 2010;5(1):e8812.

19. Pruesse E, Peplies J, Glöckner FO. SINA: accurate high-throughput multiple sequence alignment of ribosomal RNA genes. Bioinformatics. 2012;28(14):1823-9.

20. Quast C, Pruesse E, Yilmaz P, Gerken J, Schweer T, Yarza P, et al. The SILVA ribosomal RNA gene database project: improved data processing and web-based tools. Nucleic acids research. 2012:gks1219.

21. Westram R, Bader K, Prüsse E, Kumar Y, Meier H, Glöckner FO, et al. ARB: a software environment for sequence data. Handbook of Molecular Microbial Ecology I: Metagenomics and Complementary Approaches, Wiley-Blackwell. 2011:399-406.

22. Katoh K, Toh H. Recent developments in the MAFFT multiple sequence alignment program. Briefings in bioinformatics. 2008;9(4):286-98.

23. Katoh K, Asimenos G, Toh H. Multiple alignment of DNA sequences with MAFFT. Bioinformatics for DNA sequence analysis: Springer; 2009. p. 39-64.

24. Tamura K, Peterson D, Peterson N, Stecher G, Nei M, Kumar S. MEGA5: molecular evolutionary genetics analysis using maximum likelihood, evolutionary distance, and maximum parsimony methods. Molecular biology and evolution. 2011;28(10):2731-9.

25. Waterhouse AM, Procter JB, Martin DM, Clamp M, Barton GJ. Jalview Version 2—a multiple sequence alignment editor and analysis workbench. Bioinformatics. 2009;25(9):1189-91.

26. Aspeborg H, Coutinho PM, Wang Y, Brumer H, Henrissat B. Evolution, substrate specificity and subfamily classification of glycoside hydrolase family 5 (GH5). BMC Evolutionary Biology. 2012;12(1):186.

27. Petersen TN, Brunak S, von Heijne G, Nielsen H. SignalP 4.0: discriminating signal peptides from transmembrane regions. Nature methods. 2011;8(10):785-6.

28. Miller GL. Use of dinitrosalicylic acid reagent for determination of reducing sugar. Analytical chemistry. 1959;31(3):426-8.
