# Supplementary material for: Insight into Dominant Cellulolytic Bacteria from Two Biogas Digesters and Their Glycoside Hydrolase Genes
Source: PLoS One. 2015 Jun 12;10(6):e0129921. doi: 10.1371/journal.pone.0129921 (PMC4466528; doi:10.1371/journal.pone.0129921)
Supplement: S1 Table — (DOCX) [file pone.0129921.s010.docx]

**S1 Table.** Primers used for verification of GH-containing contigs assembled from the metagenomic short reads by the refined assembly method and screening of fosmid clones harboring these GH-containing or BG-1 16S rRNA gene.

| Primer name | Primer sequences (5'→3') | Design purposes |
| --- | --- | --- |
| BG-1 16SF | GCATCACATTAAGAGGAAAGGTTACG | Screening of fosmid clones harboring BG-1 16S rRNA gene |
| BG-1 16SR | TCATGCTTGCGCACGTACTCCC |  |
| Contig10356_780_2F | GGCCCAAATTGATGAACGGA | Verification of 780bp gene fragment in Contig10356 |
| Contig10356_780_2R | TATTGTGTTGCACCCGGAAC |  |
| Contig10365_803_3F | TGCCGGAAAATACGATCTGC | Verification of 803bp gene fragment in Contig10365 |
| Contig10365_803_3R | CGCAATGCCTGGTATCTCTG |  |
| Contig12561_1300_1F* | CCTATGCCAGCGAACTTGAG | Verification of 1300bp gene fragment in Contig12561 |
| Contig12561_1300_1R* | ACGGGTTCGCCGTTTTTATT |  |
| Contig12673_920_2F | ATGTCGGCTGTGGAAAACAG | Verification of 920bp gene fragment in Contig12673 |
| Contig12673_920_2R | CAACCTGTAAACGGTTCCCC |  |
| Contig12911_970_1F | TTTGACCCAGCTAAGGAGCA | Verification of 970bp gene fragment in Contig12911 |
| Contig12911_970_1R | AGGGCATCAACACCACTGTA |  |
| Contig12936_1170_1F | AAGAGGGAACACCCGTCAAT | Verification of 1170bp gene fragment in Contig12936 |
| Contig12936_1170_1R | GCAATACCCGATGCGAGAAA |  |
| Contig12975_702_1F | CAGCAAATGCCTACATGGCT | Verification of 702bp gene fragment in Contig12975 |
| Contig12975_702_1R | TAATACCGGGCTTCCCTCAC |  |
| Contig13057_1301_2F* | TGGTCAAAATCCTTCGTGGC | Verification of 1301bp gene fragment in Contig10357 |
| Contig13057_1301_2R* | ATCCGGCAATGGCACTACTA |  |
| Contig13192_747_1F* | GGCGAAGCGGGGAAAACCCA | Verification of 747bp gene fragment in Contig13192 |
| Contig13192_747_1R* | CGCCCCTAACCCCCGGTGTA |  |
| Contig13199_1025_3F | GCAGTGGTTCTGCAGATGTT | Verification of 1025bp gene fragment in Contig13199 |
| Contig13199_1025_3R | TCCTTTCGGAAATCTGCCCT |  |
| Contig13219_700_2F | ATGCGGCATCAAGGGAATTT | Verification of 700bp gene fragment in Contig13219 |
| Contig13219_700_2R | GCGGCAATCTTAGAGGTGTG |  |
| Contig13228_840_1F | GAAAACACTGGCGGATGGAA | Verification of 840bp gene fragment in Contig13228 |
| Contig13228_840_1R | TGACCCTGGAATCCAACTCC |  |
| Contig13630_864_1F | TTTTCCGGGCCAGTAGTTCT | Verification of 864bp gene fragment in Contig13630 |
| Contig13630_864_1R | GGGCATGGGCATTACAACAT |  |
| Contig13811_1200_1F | TAGCATACGGTGAAGGGACC | Verification of 1200bp gene fragment in Contig13811 |
| Contig13811_1200_1R | GCATGTCCATATAGCGTCCG |  |
| Contig13954_613_1F | GTGCTTGGGAAAGAATGGGCATGA | Verification of 613bp gene fragment in Contig13954 |
| Contig13954_613_1R | GGCTGCTGTGGCTGTCCTGG |  |
| Contig14104_1450_1F | AGCAGCACGAAAAACTGTCA | Verification of 1450bp gene fragment in Contig14104 |
| Contig14104_1450_1R | CCGTGACAGAGCTGCTTTAG |  |
| Contig16802_425_1F | GAAGCAGGCCGCTACCGCAA | Verification of 425bp gene fragment in Contig16802 |
| Contig16802_425_1R | CCTTGGCGGACGGGCAAGTT |  |
| Contig16857_858_1F | CGGCTTTTGTTTTGCCTACG | Verification of 858bp gene fragment in Contig16857 |
| Contig16857_858_1R | TATCAGATCGCTCGGTGGAG |  |
| Contig17862_1109_1F | GAACCAATGTGCAAGTTCGC | Verification of 1109bp gene fragment in Contig17862 |
| Contig17862_1109_1R | ACAGGGTTCCGACCAAATCT |  |
| Contig17867_1346_2F | AGCGCATGACCTCTTCCATA | Verification of 1346bp gene fragment in Contig17867 |
| Contig17867_1346_2R | TTGCTAAGGACAGGGAAGCA |  |
| Contig17870_1162_1F | ATCTGATGGGCAGGGAAACA | Verification of 1162bp gene fragment in Contig17870 |
| Contig17870_1162_1R | CGTTGAGAGCGTGTAAGCAT |  |
| Contig17881_982_1F | TTAAAGCCGGCAGGTTCTTG | Verification of 982bp gene fragment in Contig17881 |
| Contig17881_982_1R | TCCCGCCAATTGTTGAATCC |  |
| Contig18571_825_4F | AATATCAGTGGGCGGTTGGA | Verification of 825bp gene fragment in Contig18571 |
| Contig18571_825_4R | TCCCCGCTTAGTTCCCAAAT |  |
| Contig18770_1318_3F | TATAGTACGGCGTGGTAGCC | Verification of 1318bp gene fragment in Contig18770 |
| Contig18770_1318_3R | CGGTGGATGAACTGAACGAG |  |
| Contig18843_1200_1F | TGGGTTTCTCTGCAGGCTTA | Verification of 1200bp gene fragment in Contig18843 |
| Contig18843_1200_1R | TGGCAACTTTGGTGGAACAG |  |
| Contig18849_1120_1F | AGGCGAAATGCAACGGATAG | Verification of 1120bp gene fragment in Contig18849 |
| Contig18849_1120_1R | TAGGGGCAAAGACTGCAGAA |  |
| Contig18878_1093_1F* | ACAACATGCTGATTGCTCCC | Verification of 1093bp gene fragment in Contig18878 |
| Contig18878_1093_1R* | CGGTTGGTAAATGCCCACAT |  |
| Contig19533_1000_2F | GAGTCTGGTTCCATCCTCGT | Verification of 1000bp gene fragment in Contig19533 |
| Contig19533_1000_2R | ACTACTGTTTGTATGCGCCG |  |
| Contig19640_700_1F* | CAGTTTGCCGCCTGAAAGTA | Verification of 700bp gene fragment in Contig19640 |
| Contig19640_700_R* | GTTCGGGCGTTAAGTTCGAT |  |
| Contig19952_820_1F | CTATGCCGGCCTGGTTTATG | Verification of 820bp gene fragment in Contig19952 |
| Contig19952_820_1R | TTCCCAGTTCGACCAACTCA |  |
| Contig20055_755_1F | ATGTGGCGTCCTAAAAGTGC | Verification of 755bp gene fragment in Contig20055 |
| Contig20055_755_1R | TGGGGTGGACGTGTAAATGA |  |
| Contig20069_639_1F | TATGATGGACACAGCCTCCC | Verification of 639bp gene fragment in Contig20069 |
| Contig20069_639_1R | TACTGAGGTCTACTGCGACG |  |
| Contig20871_685_3F* | CCGTTCCCCCATATCATCCA | Verification of 685bp gene fragment in Contig20871 |
| Contig20871_685_3R* | ACGGGACATTGTGAAAGCAG |  |
| Contig21187_820_1F | ACATTCTGGCTGTTGTTCGG | Verification of 820bp gene fragment in Contig21187 |
| Contig21187_820_1R | GAAGGGAAAAACCGTGGAGG |  |
| Contig21425_730_3F* | ACCACGTTGTTGGGATTGTC | Verification of 730bp gene fragment in Contig21425 |
| Contig21425_730_3R* | CCATCCTGGACCTCACCTAC |  |
| Contig21644_830_1F | CGGATAGGCGTTTTCAACGA | Verification of 830bp gene fragment in Contig21644 |
| Contig21644_830_1R | ATGCGTGCTTCCTCCTTTTC |  |
| Contig21731_1056_1F | CTGACGGATTTATGCCGCTT | Verification of 1056bp gene fragment in Contig21731 |
| Contig21731_1056_1R | AACCTTCAGTATCTCGCCGT |  |
| Contig22081_851_1F | ACTTTGTGATCCGTTGCCTG | Verification of 851bp gene fragment in Contig22081 |
| Contig22081_851_1R | GCCGGACCTTATGAGGGTAA |  |
| Contig22333_1100_3F | GCAGTTGTTCCAATGCGTTC | Verification of 1100bp gene fragment in Contig22333 |
| Contig22333_1100_3R | CCGATCTTCGTGCTAATCCG |  |
| Contig22836_933_1F | GAGCCTGAAATGGTTTCCCC | Verification of 933bp gene fragment in Contig22836 |
| Contig22836_933_1R | AGCTTCCCTTTGAAATCGCC |  |
| Contig23105_923_1F | GCCCTTCTACCACCCGATAA | Verification of 923bp gene fragment in Contig23105 |
| Contig23105_923_1R | CCTCGTCATTGCCTGAATCC |  |
| Contig8188_746_1F | GGCCGGAAACTCGCCGACAA | Verification of 746bp gene fragment in Contig8188 |
| Contig8188_746_1R | ACTGGAACGACATCGGCGGG |  |
| Contig8330_900_1F | GGCCGAAAACGATACGGAAT | Verification of 900bp gene fragment in Contig8330 |
| Contig8330_900_1R | TGAGGTTGCCAATGAGGAGT |  |
| Contig8411_895_F* | GTGGTGTGGTCTTTCAGGTG | Verification of 895bp gene fragment in Contig8411 |
| Contig8411_895_R* | CTCAGCTGCAATGCTTGGAT |  |
| Contig8512_828_1F* | GTCTCCTGCATCATGCCATC | Verification of 828bp gene fragment in Contig8512 |
| Contig8512_828_1R* | TCTTCTAGGTCAAGGCGTCC |  |
| Contig8557_521_1F | GCGGCAACCAGTGCCGGTAT | Verification of 521bp gene fragment in Contig8557 |
| Contig8557_521_1R | CCGCTTGGCAGCCTTGTGGA |  |
| Contig8705_726_F* | GAGCGCACCGTGTCGACGAA | Verification of 726bp gene fragment in Contig8705 |
| Contig8705_726_R* | CAGTGAGCCGGTGGATGCCG |  |
| Contig8998_1039_1F* | ACTCCGATTCGTTCATAACATCCT | Verification of 1039bp gene fragment in Contig8998 |
| Contig8998_1039_1R* | GAAGGGACGTTGAAATATATGAAC |  |
| Contig9692_606_1F | TTATCCAGGTGGACAGTCGG | Verification of 606bp gene fragment in Contig9692 |
| Contig9692_606_1R | CTTTGGGATTTTCCCCCGTC |  |
| Contig9828_1500_1F* | GAGTCTTGCCGGAGGTTAGA | Verification of 1500bp gene fragment in Contig9828 |
| Contig9828_1500_1R* | GTCCCCCAACCAAATAACCG |  |

*These primer pairs were also used for screening of fosmid clones harboring GH-containing contigs.
