# Supplementary material for: Insight into Dominant Cellulolytic Bacteria from Two Biogas Digesters and Their Glycoside Hydrolase Genes
Source: PLoS One. 2015 Jun 12;10(6):e0129921. doi: 10.1371/journal.pone.0129921 (PMC4466528; doi:10.1371/journal.pone.0129921)
Supplement: S2 Table — (DOCX) [file pone.0129921.s011.docx]

**Table S2.** Strains and plasmids used in this study.

|  | Relevant characteristics | Source or reference |
| --- | --- | --- |
| **Strains** |  |  |
| EPI-300 | Cloning host | Epicentre |
| BL21 | Expression host | Novagen |
| **Plasmids** |  |  |
| pET-28a | Expression vector, Km^r^ | Novagen |
| pET-28a-Cel1 | pET-28a harboring Cel1 gene | This work |
| pET-28a-Cel2 | pET-28a harboring Cel2 gene | This work |
| pET-28a-Cel3 | pET-28a harboring Cel3 gene | This work |
| pET-28a-Cel4 | pET-28a harboring Cel4 gene | This work |
| pET-28a-Cel5^1^ | pET-28a harboring Cel5 gene | This work |
| pET-28a-Cel6^1^ | pET-28a harboring Cel6 gene | This work |

^1^Cel5 and Cel6 were also respectively named as man1 and en2 in elsewhere (Yan *et al*., 2013).

Reference

1. Yan X, Geng A, Zhang J, Wei Y, Zhang L, Qian C, et al. Discovery of (hemi-) cellulase genes in a metagenomic library from a biogas digester using 454 pyrosequencing. Applied Microbiology and Biotechnology. 2013;97(18):8173-82.
