# Supplementary material for: Insight into Dominant Cellulolytic Bacteria from Two Biogas Digesters and Their Glycoside Hydrolase Genes
Source: PLoS One. 2015 Jun 12;10(6):e0129921. doi: 10.1371/journal.pone.0129921 (PMC4466528; doi:10.1371/journal.pone.0129921)
Supplement: S3 Table — (DOCX) [file pone.0129921.s012.docx]

**S3 Table.** Primers used for cloning and expression of six GH5 family genes in this study.

| Primers | Design purposes | Primer sequences (5′→3′) |
| --- | --- | --- |
| Cel1_F | Cloning and expression of gene Cel1 | CCGGAATTCATGGCTGGTGAACGTATAAGAATC |
| Cel1_R |  | CCGCTCGAGAGAAGGCTCTCCTTGCGACTTCCTT |
| Cel2_F | Cloning and expression of gene Cel2 | CCGGAATTCATGAAGAAAATATTTATTC |
| Cel2_R |  | CCGCTCGAGAAATATTTTTATTTTTTAC |
| Cel3_F | Cloning and expression of gene Cel3 | CCGGAATTCTTGGATGCTGATGGTAAAGC |
| Cel3_R |  | CCGCTCGAGAATTGAGTTTTTCTTTAAAAAG |
| Cel4_F | Cloning and expression of gene Cel4 | CCGGAATTCATGGCCAGATGGAGGATAAGATCG |
| Cel4_R |  | CCGCTCGAGAAGGTCTCTGAGCGTATTTCATCAT |
| Cel5_F | Cloning and expression of gene Cel5 | CGCGGAGCTCGAAGGGACGTTGAAATATATGAAC |
| Cel5_R |  | GCCGCTCGAGACTCCGATTCGTTCATAACATCCT |
| Cel6_F | Cloning and expression of gene Cel6 | CGCGGAGCTCTTGTCCAATAATGCATTTGGTTATG |
| Cel6_R |  | GCCGCTCGAGAAGGCATTCCCGTAACTTTAACCCCA |

Restriction sites are underlined.
