# Supplementary material for: Insight into Dominant Cellulolytic Bacteria from Two Biogas Digesters and Their Glycoside Hydrolase Genes
Source: PLoS One. 2015 Jun 12;10(6):e0129921. doi: 10.1371/journal.pone.0129921 (PMC4466528; doi:10.1371/journal.pone.0129921)
Supplement: S4 Table — (DOCX) [file pone.0129921.s013.docx]

**S4 Table.** Diversity of microbial communities of Z7 and Z8 based on analysis of 16S rRNA gene clone libraries.

(a) Diverstiy of Bacteria

|  | Z7 | Z8 | All |
| --- | --- | --- | --- |
| Sequences | 775 | 604 | 1379 |
| Shannon | 4.456 | 4.049 | 4.761 |
| Simpson | 0.976 | 0.963 | 0.981 |
| Observed OTU | 225 | 151 | 339 |
| Singletons | 157 | 89 | 216 |
| Good coverage index | 0.8 | 0.85 | 0.84 |

(b) Diverstiy of Archaea

|  | Z7 | Z8 | All |
| --- | --- | --- | --- |
| Sequences | 89 | 78 | 167 |
| Shannon | 1.873 | 1.362 | 1.783 |
| Simpson | 0.796 | 0.684 | 0.785 |
| Observed OTU | 14 | 8 | 15 |
| Singletons | 8 | 4 | 8 |
| Good coverage index | 0.91 | 0.95 | 0.95 |
