# Supplementary material for: Insight into Dominant Cellulolytic Bacteria from Two Biogas Digesters and Their Glycoside Hydrolase Genes
Source: PLoS One. 2015 Jun 12;10(6):e0129921. doi: 10.1371/journal.pone.0129921 (PMC4466528; doi:10.1371/journal.pone.0129921)
Supplement: S5 Table — (DOCX) [file pone.0129921.s014.docx]

**S5 Table.** Phylogenetic distribution of 16S rRNA genes from clone libraries and metagenomic data.

(a) Phylogenetic distribution of Bacteria.

| Taxonomy | Z7-PCR^1^ | Z7-meta^2^ | Z8-PCR^1^ | Z8-meta^2^ |
| --- | --- | --- | --- | --- |
| *Proteobacteria* | 4.26% | 4.75% | 6.79% | 7.91% |
| *Gemmatimonadetes* | 0.00% | 0.32% | 0.00% | 0.00% |
| *LCP-26* | 0.00% | 0.32% | 0.00% | 0.00% |
| *Bacteroidetes* | 20.77% | 10.44% | 37.42% | 31.75% |
| *Verrucomicrobia* | 0.52% | 0.63% | 1.82% | 2.31% |
| *Lentisphaerae* | 0.00% | 0.00% | 0.33% | 0.36% |
| *WS3* | 0.00% | 0.00% | 0.00% | 0.24% |
| *Haloanaerobiales* | 0.00% | 0.16% | 0.00% | 0.12% |
| *Actinobacteria* | 0.13% | 1.27% | 0.00% | 0.97% |
| *Spirochaetes* | 0.39% | 2.69% | 4.30% | 12.29% |
| *Fibrobacteres* | 0.00% | 0.00% | 0.00% | 0.61% |
| *Chloroflexi* | 0.13% | 0.32% | 0.00% | 0.12% |
| *JS1* | 0.00% | 0.00% | 0.00% | 0.24% |
| *Natronoanearobium* | 0.52% | 0.79% | 0.66% | 1.22% |
| *Firmicutes* | 72.77% | 77.06% | 48.68% | 41.12% |
| *Thermoanaerobacteriales* | 0.26% | 0.00% | 0.00% | 0.00% |
| *Aminanaerobia* | 0.13% | 0.79% | 0.00% | 0.73% |
| Unclassified bacteria | 0.13% | 0.47% | 0.00% | 0.00% |
| Number of bacteria 16S rRNA gene sequence | 775 | 632 | 604 | 822 |

(b) Phylogenetic distribution of archaea.

| Taxonomy | Z7-PCR^1^ | Z7-meta^2^ | Z8-PCR^1^ | Z8-meta^2^ |
| --- | --- | --- | --- | --- |
| *Methanomicrobia* | 76.40% | 87.50% | 91.03% | 83.33% |
| *Methanosphaera* | 23.60% | 12.50% | 8.97% | 16.67% |
| Number of archaeal 16S rRNA gene sequence | 89 | 8 | 78 | 24 |

^1^Proportion of 16S rRNA gene sequences in the 16S rRNA clone libraries of Z7 and Z8.

^2^ Proportion of metagenomic reads annotated as16S rRNA genes.
