# Supplementary material for: Insight into Dominant Cellulolytic Bacteria from Two Biogas Digesters and Their Glycoside Hydrolase Genes
Source: PLoS One. 2015 Jun 12;10(6):e0129921. doi: 10.1371/journal.pone.0129921 (PMC4466528; doi:10.1371/journal.pone.0129921)
Supplement: S6 Table — (DOCX) [file pone.0129921.s015.docx]

**S6 Table.** Overview of sequence annotation of metagenomes.

|  | Z7 | Z8 |
| --- | --- | --- |
| Reads number | 519,015 | 668,860 |
| Base (Mb) | 122.46 | 158.54 |
| Medium read length(bp) | 253 | 254 |
| Average read length(bp) | 235.9 | 237 |
| NR hits | 360,433 (69.45%)^1^ | 461,329 (68.97%) |
| Taxonomy assigned^2^ | 357,362 (68.85%) | 458,225 (68.51%) |
| COG hits | 322,001 (62.04%) | 413,186 (61.77%) |
| COG assigned^3^ | 292,788 (56.41%) | 374,585 (56.00%) |
| KO hits | 324,056 (62.44%) | 446,343 (66.73%) |
| KO assigned^3^ | 212,170 (40.88%) | 279,929 (41.85%) |
| RDP hits | 807 (0.16%) | 1,014 (0.15%) |
| 16S rRNA^3^ | 640(0.12%) | 846(0.13%) |

^1^Data indicated in the parentheses is relative abundance, divided by the number of total sequenced reads in the metagenome.

^2^Taxonomy assigned by MEGAN based on NR hits.

^3^Annotated metagenomic reads defined by the thresholds were described in the supporting information S1.
