# Supplementary material for: Insight into Dominant Cellulolytic Bacteria from Two Biogas Digesters and Their Glycoside Hydrolase Genes
Source: PLoS One. 2015 Jun 12;10(6):e0129921. doi: 10.1371/journal.pone.0129921 (PMC4466528; doi:10.1371/journal.pone.0129921)
Supplement: S7 Table — (DOCX) [file pone.0129921.s016.docx]

**S7 Table.** Dominant species^1^ identified by MEGAN analysis from both metagenomes.

| Species name | Function | Z7^2^ | Z8^2^ | Z7_R^3^ | Z8_R^3^ |
| --- | --- | --- | --- | --- | --- |
| *Clostridium thermocellum** | Cellulolytic | 18349 | 2400 | 3.54% | 0.36% |
| *Clostridium ultunense** | Syntrophic | 7067 | 3602 | 1.36% | 0.54% |
| *Syntrophomonas wolfei** | Syntrophic | 5979 | 3020 | 1.15% | 0.45% |
| *Clostridium cellulolyticum** | Cellulolytic | 3569 | 1292 | 0.69% | 0.19% |
| *Desulfitobacterium hafniense* | Dehalogenation | 2551 | 1994 | 0.49% | 0.30% |
| *Halothermothrix orenii** | Hydrogen | 1661 | 1010 | 0.32% | 0.15% |
| *Pelotomaculum thermopropionicum** | Syntrophic | 1305 | 608 | 0.25% | 0.09% |
| *Symbiobacterium thermophilum** | Syntrophic | 1149 | 501 | 0.22% | 0.07% |
| *Caldicellulosiruptor saccharolyticus** | Cellulolytic | 1150 | 558 | 0.22% | 0.08% |
| *Moorella thermoacetica** | Homoacetogen | 1016 | 407 | 0.20% | 0.06% |
| *Clostridium leptum* | Acidogen | 1350 | 903 | 0.26% | 0.14% |
| *Alkaliphilus metalliredigens* | Metal-reducing | 1881 | 1700 | 0.36% | 0.25% |
| *Chlorobium phaeobacteroides* | Unknown | 627 | 1514 | 0.12% | 0.23% |
| *Methanospirillum hungatei* | Methanogen | 469 | 1348 | 0.09% | 0.20% |
| *Bacteroides vulgatus** | Starch hydrolysis | 435 | 1382 | 0.08% | 0.21% |
| *Bacteroides fragilis* | Acidogen | 424 | 1381 | 0.08% | 0.21% |
| *Parabacteroides merdae** | Acidogen | 319 | 1300 | 0.06% | 0.19% |
| *Bacteroides ovatus** | Acidogen | 436 | 1455 | 0.08% | 0.22% |
| *Parabacteroides distasonis* | Acidogen | 596 | 1678 | 0.11% | 0.25% |
| *Victivallis vadensis** | Hydrogen | 284 | 1362 | 0.05% | 0.20% |
| *Bacteroides uniformis** | Hydrogen | 509 | 1704 | 0.10% | 0.25% |
| *Acholeplasma laidlawii** | Unknown | 338 | 2558 | 0.07% | 0.38% |
| *Treponema denticola** | Proteolytic | 1029 | 4710 | 0.20% | 0.70% |
| *Methanoculleus marisnigri** | Methanogen | 16324 | 39944 | 3.15% | 5.97% |

^1^Species were selected for those with matched reads number ≥ 1000 in Z7 or Z8 metagenome.

^2^Absolute count of metagenomic reads from the metagenome.

^3^Relative abundance. The calculation was the assigned read number to one species divided by the number of total sequenced reads of Z7 or Z8 metagnome.

*Significant different between Z7 and Z8, tested by XIPE with 98% confidence level.
