# Supplementary material for: Insight into Dominant Cellulolytic Bacteria from Two Biogas Digesters and Their Glycoside Hydrolase Genes
Source: PLoS One. 2015 Jun 12;10(6):e0129921. doi: 10.1371/journal.pone.0129921 (PMC4466528; doi:10.1371/journal.pone.0129921)
Supplement: S8 Table — (DOCX) [file pone.0129921.s017.docx]

**S8 Table.** Metagenomic reads encoding glycoside hydrolase genes.

| CAZy family | Known activity | Pfam domain^1^ | Z7  Read number^2^ | Z8^2^  Read number^2^ |
| --- | --- | --- | --- | --- |
| GH5 | cellulase | PF00150 | 128 | 70 |
| GH9 | endoglucanase&exoglucanase | PF00759 | 241 | 51 |
| GH44 | endoglucanase& xyloglucanase | NA | 51 | 0 |
| GH45 | endoglucanase | PF02015 | 0 | 1 |
| GH48 | endo-processive cellulases | PF02011 | 27 | 2 |
| GH74 | endoglucanases & xyloglucanases | NA | 102 | 25 |
| GH8 | endo-xylanases | PF02011 | 19 | 2 |
| GH10 | endo-1,4-β-xylanase | PF00331 | 245 | 136 |
| GH11 | xylanase | PF00457 | 48 | 13 |
| GH26 | β-mannanase & xylanase | PF02156 | 31 | 10 |
| GH28 | galacturonases | PF00295 | 4 | 6 |
| GH16 | xyloglucanases & xyloglycosyltransferases | PF00722 | 25 | 29 |
| GH81 | 1,3-β-glucanase | PF03639 | 4 | 1 |
| GH51 | α-L-arabinofuranosidase | NA | 193 | 127 |
| GH54 | α-L-arabinofuranosidase | PF09206 | 0 | 2 |
| GH67 | α-glucuronidase | PF07477, PF07488 | 7 | 7 |
| GH78 | α-L-rhamnosidase | PF05592 | 64 | 190 |
| GH1 | β-glucosidase and many other β-linked dimers | PF00232 | 122 | 94 |
| GH2 | β-galactosidases and other β-linked dimers | PF02836, PF00703, PF02837 | 40 | 71 |
| GH3 | mainly β-glucosidases | PF00933 | 236 | 253 |
| GH29 | α-L-fucosidase | PF01120 | 43 | 77 |
| GH95 | α-L-fucosidase | NA | 149 | 131 |
| GH35 | β-galactosidase | PF01301 | 14 | 5 |
| GH38 | α-mannosidase | PF01074, PF07748 | 18 | 28 |
| GH39 | β-xylosidase | PF01229 | 55 | 16 |
| GH42 | β-galactosidase | PF02449, PF08533, PF08532 | 28 | 18 |
| GH4 | β-galactosidase, α-glucosidase,α-glucuronidase | PF02056.7 | 168 | 120 |
| GH13 | α-amylase |  | 147 | 251 |
| GH57 | α-amylase |  | 97 | 99 |
| GH77 | 4-α-glucanotransferase |  | 110 | 287 |
| GH97 | α-glucosidase | NA | 45 | 147 |
| GH65 | maltose phosphorylase & Kojibiose phosphorylase |  | 110 | 81 |
| GH94 | cellobiose/cellodextrin-phosphorylase |  | 533 | 279 |
| Chitin-degrading enzymes | | | | |
| GH18 | Chitinase |  | 92 | 73 |
| GH19 | Chitinase |  | 0 | 3 |
| GH14 | β-amylase (EC 3.2.1.2 ) | PF01373.8 | 1 | 0 |
| GH15 | glucoamylase (EC 3.2.1.3 ); glucodextranase (EC 3.2.1.70 ); α, α-trehalase (EC 3.2.1.28 ) | PF00723.12 | 17 | 24 |
| GH20 | β-hexosaminidase (EC 3.2.1.52 ); lacto-N-biosidase (EC 3.2.1.140 ); β-1,6-N-acetylglucosaminidase) (EC 3.2.1.-) | PF00728.13 | 82 | 90 |
| GH22 | lysozyme |  | 0 | 2 |
| GH23 | lysozyme |  | 51 | 53 |
| GH25 | lysozyme |  | 39 | 26 |
| GH27 | α-galactosidase (EC 3.2.1.22 ); α-N-acetylgalactosaminidase (EC 3.2.1.49 ); isomalto-dextranase (EC 3.2.1.94 ). | PF02065.9 | 34 | 53 |
| GH30 | O-Glycosyl hydrolyses |  | 3 | 5 |
| GH31 | α-glucosidase (EC 3.2.1.20 ); α-1,3-glucosidase (EC 3.2.1.84 ); sucrase-isomaltase (EC 3.2.1.48 ) (EC 3.2.1.10 ); α-xylosidase (EC 3.2.1.-); α-glucan lyase (EC 4.2.2.13 ); isomaltosyltransferase (EC 2.4.1.-). | PF01055.17 | 243 | 286 |
| GH32 | invertase (EC 3.2.1.26 ); inulinase (EC 3.2.1.7 ); 2,6-β-fructan 6-levanbiohydrolase (EC 3.2.1.64 ); levanase (EC 3.2.1.65 ); exo-inulinase (EC 3.2.1.80 ); sucrose:sucrose 1-fructosyl transferase (EC 2.4.1.99 ); fructan fructan 1-fructosyltransferase (EC 2.4.1.100 ); fructan β-(2,1)-fructosidase (EC 3.2.1.153 ); fructan β-(2,6)-fructosidase (EC 3.2.1.154 ). | PF00251.11 | 15 | 19 |
| GH36 | α-galactosidase (EC 3.2.1.22 ); α-N-acetylgalactosaminidase (EC 3.2.1.49 ); stachyose synthase (EC 2.4.1.67 ); raffinose synthase (EC 2.4.1.82 ) |  | 21 | 39 |
| GH37 | α, α-trehalase (EC 3.2.1.28 ). | PF01204.9 | 1 | 1 |
| GH50 | β-agarase |  | 4 | 23 |
| GH55 | exo-1,3-glucanase (EC 3.2.1.58 ); endo-1,3-glucanase (EC 3.2.1.39 ). |  | 4 | 6 |
| GH59 | galactocerebrosidase (EC 3.2.1.46 ) | PF02057.6 | 1 | 0 |
| GH66 | cycloisomaltooligosaccharide glucanotransferase (EC 2.4.1.- ); dextranase (EC 3.2.1.11 ). |  | 1 | 2 |
| GH73 | Mannosyl-glycoprotein endo-β-N-acetylglucosaminidase | PF01832.11 | 32 | 63 |
| GH76 | α-1,6-mannanase (EC 3.2.1.101 ) | PF03663.5 | 0 | 3 |
| GH82 | carrageenase |  | 0 | 2 |
| GH87 | mycodextranase (EC 3.2.1.61 ); α-1,3-glucanase (EC 3.2.1.59 ) |  | 1 | 0 |
| GH88 | Glycosyl Hydrolase Family 88 | PF07470.4 | 13 | 53 |
| GH89 | α-N-acetylglucosaminidase (NAGLU) | PF05089.3 | 0 | 3 |
| GH93 | exo-1,5-α-L-arabinanase (EC 3.2.1.-) |  | 1 | 0 |
| GH99 | Glycoprotein endo-α-1,2-mannosidase (EC 3.2.1.130 ) |  | 0 | 2 |
| GH101 | endo-α-N-acetylgalactosaminidase (EC 3.2.1.97 ) |  | 2 | 2 |
| GH102 | Peptidoglycan lytic transglycosylase |  | 1 | 6 |
| GH103 | peptidoglycan lytic transglycosylase (EC 3.2.1.- ) |  | 8 | 22 |
| GH104 | Peptidoglycan lytic transglycosylase |  | 1 | 2 |
| GH106 | α-L-rhamnosidase (EC 3.2.1.40 ) |  | 13 | 47 |
| GH107 | sulfated fucan endo-1,4-fucanase (EC 3.2.1.- ) |  | 18 | 7 |
| GH109 | α-N-acetylgalactosaminidase (EC 3.2.1.49 ) |  | 44 | 154 |
| GH110 | α-galactosidase (EC3.2.1.22); α-1,3-galactosidase (EC 3.2.1.-) |  | 3 | 3 |

^1^Pfam database ID (http://pfam.sanger.ac.uk/) for the corresponding GH families.

^2^Number of metagenomic reads which were annotated as GH family genes.
