# Supplementary material for: Insight into Dominant Cellulolytic Bacteria from Two Biogas Digesters and Their Glycoside Hydrolase Genes
Source: PLoS One. 2015 Jun 12;10(6):e0129921. doi: 10.1371/journal.pone.0129921 (PMC4466528; doi:10.1371/journal.pone.0129921)
Supplement: S9 Table — (DOCX) [file pone.0129921.s018.docx]

**S9 Table.** Metagenomic reads encoding CBM identified in the Z7 and Z8 metagenomes.

| Pfam HMM name | CAZy family | Known function | Z7 | Z8 |
| --- | --- | --- | --- | --- |
| PF03424.5 | CBM17/28 | Amorphous cellulose/crystalline cellulose binding domain | 6 | 1 |
| PF00553.10 | CBM2 | Cellulose-, chitin- and xylan binding domain. | 9 | 4 |
| PF00686.10 | CBM20 | Granular starch-binding domain | 0 | 4 |
| PF00942.9 | CBM3 | Cellulose- and chitin-binding domain | 54 | 4 |
| PF02018.8 | CBM4/9 | Cellulose- and xylan-binding domain | 38 | 16 |
| PF02839.5 | CBM5/12 | Chitin-binding domain | 0 | 2 |
| PF03422.6 | CBM6 | Amorphous cellulose- and xylan-binding domain | 166 | 74 |
|  | CBM30 | Cellulose-binding domain | 7 | 10 |
|  | CBM35 | Granular starch-binding domain | 2 | 0 |
|  | CBM36 | Xylan-banding domain | 65 | 34 |
|  | CBM37 | Broad binding specificity | 11 | 0 |
|  | CBM41 | α-glucans amylose-, amylopectin-, pullulan-binding domain | 33 | 20 |
|  | CBM42 | Arabinofuranose-binding domain | 3 | 0 |
|  | CBM44 | Cellulose- and xylan-binding domain | 6 | 0 |
|  | CBM46 | Cellulose-binding domain | 24 | 7 |
|  | CBM48 | Glycogen-binding domain | 3 | 0 |
