# Supplementary material for: Insight into Dominant Cellulolytic Bacteria from Two Biogas Digesters and Their Glycoside Hydrolase Genes
Source: PLoS One. 2015 Jun 12;10(6):e0129921. doi: 10.1371/journal.pone.0129921 (PMC4466528; doi:10.1371/journal.pone.0129921)
Supplement: S10 Table — (DOCX) [file pone.0129921.s019.docx]

**S10 Table.** Metagenomic reads encoding cellulosomal genes in Z7 and Z8 metagenomes.

| Gene products | Gene ID | Organisms | Z7^1^ | Z8^1^ |
| --- | --- | --- | --- | --- |
| Cellulosome anchoring protein, cohesin region | gi\|118726845 | *Clostridium cellulolyticum* H10 | 0 | 1 |
|  | gi\|118726850 | *Clostridium cellulolyticum* H10 | 23 | 10 |
|  | gi\|125972973 | *Clostridium thermocellum* ATCC 27405 | 16 | 3 |
|  | gi\|125973254 | *Clostridium thermocellum* ATCC 27405 | 37 | 2 |
|  | gi\|125973822 | *Clostridium thermocellum* ATCC 27405 | 69 | 30 |
|  | gi\|125975556 | *Clostridium thermocellum* ATCC 27405 | 102 | 4 |
|  | gi\|125975557 | *Clostridium* *thermocellum* ATCC 27405 | 114 | 21 |
|  | gi\|125975558 | *Clostridium* *thermocellum* ATCC 27405 | 64 | 18 |
|  | gi\|125975559 | *Clostridium* *thermocellum* ATCC 27405 | 50 | 10 |
|  | gi\|125973253 | *Clostridium* *thermocellum* ATCC 27405 | 3 | 0 |
|  | gi\|150398888 | *Methanococcus* *vannielii* SB | 3 | 1 |
|  | gi\|169186289 | *Paenibacillus* sp. JDR-2 | 38 | 12 |
|  | gi\|163799418 | *Methanococcus* *voltae* A3 | 5 | 4 |
| Cellulosome enzyme, dockerin type I | gi\|167588077 | *Burkholderia* *ubonensis* Bu | 1 | 1 |
|  | gi\|182420335 | *Clostridium* *butyricum* 5521 | 10 | 5 |
|  | gi\|118725649 | *Clostridium cellulolyticum* H10 | 34 | 39 |
|  | gi\|118725686 | *Clostridium cellulolyticum* H10 | 2 | 1 |
|  | gi\|118726507 | *Clostridium* *cellulolyticum* H10 | 2 | 0 |
|  | gi\|118726750 | *Clostridium* *cellulolyticum* H10 | 1 | 0 |
|  | gi\|118726827 | *Clostridium* *cellulolyticum* H10 | 63 | 47 |
|  | gi\|118726842 | *Clostridium* *cellulolyticum* H10 | 6 | 2 |
|  | gi\|118726973 | *Clostridium* *cellulolyticum* H10 | 22 | 8 |
|  | gi\|118727345 | *Clostridium* *cellulolyticum* H10 | 31 | 4 |
|  | gi\|118727951 | *Clostridium* *cellulolyticum* H10 | 16 | 5 |
|  | gi\|118727971 | *Clostridium* *cellulolyticum* H10 | 7 | 4 |
|  | gi\|118728085 | *Clostridium* *cellulolyticum* H10 | 48 | 51 |
|  | gi\|118728086 | *Clostridium* *cellulolyticum* H10 | 48 | 4 |
|  | gi\|125972568 | *Clostridium* *thermocellum* ATCC 27405 | 14 | 4 |
|  | gi\|125972761 | *Clostridium* *thermocellum* ATCC 27405 | 112 | 85 |
|  | gi\|125972780 | *Clostridium* *thermocellum* ATCC 27405 | 55 | 26 |
|  | gi\|125972956 | *Clostridium* *thermocellum* ATCC 27405 | 5 | 0 |
|  | gi\|125972959 | *Clostridium* *thermocellum* ATCC 27405 | 3 | 0 |
|  | gi\|125973158 | *Clostridium* *thermocellum* ATCC 27405 | 51 | 1 |
|  | gi\|125973247 | *Clostridium* *thermocellum* ATCC 27405 | 61 | 4 |
|  | gi\|125973435 | *Clostridium* *thermocellum* ATCC 27405 | 4 | 4 |
|  | gi\|125973912 | *Clostridium* *thermocellum* ATCC 27405 | 33 | 4 |
|  | gi\|125974310 | *Clostridium* *thermocellum* ATCC 27405 | 8 | 6 |
|  | gi\|125974394 | *Clostridium* *thermocellum* ATCC 27405 | 14 | 20 |
|  | gi\|125974530 | *Clostridium* *thermocellum* ATCC 27405 | 3 | 1 |
|  | gi\|125974624 | *Clostridium* *thermocellum* ATCC 27405 | 28 | 8 |
|  | gi\|125974756 | *Clostridium* *thermocellum* ATCC 27405 | 2 | 0 |
|  | gi\|125975033 | *Clostridium* *thermocellum* ATCC 27405 | 1 | 0 |
|  | gi\|125975360 | *Clostridium* *thermocellum* ATCC 27405 | 10 | 0 |
|  | gi\|125975610 | *Clostridium* *thermocellum* ATCC 27405 | 30 | 0 |

^1^Number of metagenomic reads which have hits with e-value ≤1.0E-5 in the BLASTX against reference cellulosomal genes.
