# Supplementary material for: Insight into Dominant Cellulolytic Bacteria from Two Biogas Digesters and Their Glycoside Hydrolase Genes
Source: PLoS One. 2015 Jun 12;10(6):e0129921. doi: 10.1371/journal.pone.0129921 (PMC4466528; doi:10.1371/journal.pone.0129921)
Supplement: S11 Table — (DOCX) [file pone.0129921.s020.docx]

**S11 Table.** Numbers of reads or contigs in the GH-containing contigs recovering process with the refinery assembly approach in this study.

|  | Reads or contigs numbers |
| --- | --- |
| Total Reads number in Z7 and Z8 | 1,187,875 |
| Targeted reads number | 44,477 |
| Total assembled contigs | 7438 |
| Contigs ≥ 1Kb | 219 |
| GH-containing contigs | 163 |
| Full-length GH genes | 33 |
| Targeted GH-containing contigs | 41 |
