# Supplementary material for: Insight into Dominant Cellulolytic Bacteria from Two Biogas Digesters and Their Glycoside Hydrolase Genes
Source: PLoS One. 2015 Jun 12;10(6):e0129921. doi: 10.1371/journal.pone.0129921 (PMC4466528; doi:10.1371/journal.pone.0129921)
Supplement: S12 Table — (DOCX) [file pone.0129921.s021.docx]

**S12 Table.** The 163 GH-containing contigs assembled from the metagenomic data of biogas digesters with the refined assembly approach.

| Contig ID  (Length, bp)^1^ | BD_contig ID  (Length, bp)^2^ | CAZymer in each contigs | Z7 number^3^ | Z8 number^3^ | Depth |
| --- | --- | --- | --- | --- | --- |
| Contig17867  (14950) | BD_contig00038 (6927) | GH13 | 75 | 362 | 7.42 |
| Contig17870  (13825) | BD_contig00051 (6254) | GH95 | 74 | 388 | 8.48 |
| Contig19269  (10476) | BD_contig00111  (4634) | GH53 | 50 | 288 | 8.19 |
| Contig18491  (9404) | BD_contig00114  (4608) | GH92 | 80 | 364 | 11.98 |
| Contig19952  (8479) | BD_contig00263  (3229) | GH4,GH43 | 125 | 85 | 6.28 |
| Contig18770  (8386) | BD_contig00061  (5824) | GH13 | 31 | 191 | 6.72 |
| Contig17008  (7343) | BD_contig00040  (6832) | GH2 | 38 | 169 | 7.16 |
| Contig12673  (6557) | BD_contig00225  (3409) | GH43-CBM6, α-L-arabinofuranosidase | 320 | 154 | 18.31 |
| Contig20871  (5841) | BD_contig00148  (4111) | GH9 | 19 | 145 | 7.13 |
| Contig22333  (5626) | BD_contig00550  (2523) | GH18-CBM6 | 152 | 5 | 7.06 |
| Contig13192  (5397) | BD_contig00728  (2096) | GH10-GH43-CBM6,GH39,GH10 | 80 | 17 | 4.55 |
| Contig16857  (5124) | BD_contig00807 (2004) | GH2 | 63 | 31 | 4.65 |
| Contig13630  (5085) | BD_contig01193  (1738) | GH10-CBM_4_9 | 71 | 26 | 4.83 |
| Contig12936  (4827) | BD_contig00305  (3005) | GH43-CBM6 | 123 | 176 | 15.71 |
| Contig17364  (4112) | BD_contig00155  (4042) | GH97 | 18 | 105 | 7.59 |
| Contig20473  (3952) | BD_contig00360  (2816) | GH18-CBM6,GH25 | 52 | 48 | 6.41 |
| Contig12561  (3716) | BD_contig00181  (3692) | GH3 | 58 | 18 | 5.18 |
| Contig20677  (3607) | BD_contig00452  (2587) | GH13 | 9 | 78 | 6.12 |
| Contig17881  (3528) | BD_contig02115 (1353) | GH31 | 242 | 110 | 25.27 |
| Contig17485  (3473) | BD_contig00219  (3446) | GH2 | 22 | 79 | 7.38 |
| Contig12911  (3412) | BD_contig03008(1152) | GH3 | 328 | 106 | 32.21 |
| Contig16802  (3391) | BD_contig01208  (1729) | GH5,GH8 | 51 | 16 | 5 |
| Contig17914  (3381) | BD_contig00649  (2178) | GH94-CBMX | 48 | 11 | 4.42 |
| Contig8330  (3378) | BD_contig00369  (2795) | GH5,GH31 | 52 | 13 | 4.87 |
| Contig19140  (3222) | BD_contig03558  (2976) | GH18 | 12 | 87 | 7.8 |
| Contig17862  (3155) | BD_contig00680  (2146) | GH2-CBM6 | 43 | 14 | 4.58 |
| Contig13057  (3111) | BD_contig02431  (1266) | GH10,GH43,GH95 | 40 | 11 | 4.15 |
| Contig20598  (3005) | BD_contig00304  (3005) | GH65 | 39 | 16 | 4.64 |
| Contig19219  (2934) | BD_contig00491  (2493) | GH2 | 44 | 11 | 4.75 |
| Contig8557  (2860) | BD_contig02912  (1164) | GH5 | 13 | 24 | 3.28 |
| Contig19025  (2737) | BD_contig03558  (1060) | GH65 | 36 | 0 | 3.33 |
| Contig21731  (2659) | BD_contig02771  (1193) | GH43-CBM6 | 18 | 31 | 4.67 |
| Contig18961  (2587) | BD_contig00739  (2074) | GH77,GH57 | 6 | 59 | 6.38 |
| Contig18843  (2535) | BD_contig03613  (1053) | GH43,GH95 | 32 | 10 | 4.2 |
| Contig19910  (2479) | BD_contig00503  (2457) | GH37 | 45 | 0 | 4.59 |
| Contig9828  (2463) | BD_contig01257  (1702) | GH9-CBM6 | 21 | 11 | 3.29 |
| Contig13199  (2454) | BD_contig01793  (1449) | GH11-CBM6-dockerin | 62 | 2 | 6.6 |
| Contig16822  (2438) | BD_contig03704 (1035) | GH94 | 30 | 9 | 4.05 |
| Contig17475  (2438) | BD_contig01581  (1528) | GH18 | 37 | 5 | 4.36 |
| Contig12569  (2351) | ND | GH3 | 25 | 17 | 4.53 |
| Contig13099  (2309) | ND | GH43-CBM6-dockerin | 35 | 0 | 3.83 |
| Contig14104  (2297) | ND | GH3,GH5 | 28 | 0 | 3.08 |
| Contig20338  (2229) | BD_contig00685  (2142) | GH31 | 32 | 22 | 6.14 |
| Contig17985  (2212) | ND | GH109 | 0 | 29 | 3.33 |
| Contig22081  (2181) | ND | GH3,GH43 | 20 | 4 | 2.79 |
| Contig19881  (2180) | BD_contig01376  (1629) | GH94 | 16 | 12 | 3.26 |
| Contig19600  (2153) | BD_contig03376  (1086) | GH8 | 30 | 0 | 3.53 |
| Contig21331  (2144) | ND | α-amylase | 2 | 19 | 2.49 |
| Contig18033  (2097) | BD_contig02847 (1182) | GH94 | 1 | 27 | 3.39 |
| Contig20311  (2095) | ND | GH2 | 22 | 17 | 4.72 |
| Contig20261  (2074) | ND | GH81 | 23 | 0 | 2.81 |
| Contig20669  (2050) | ND | GH67 | 18 | 5 | 2.84 |
| Contig13228  (2022) | ND | GH10-CBM6 | 35 | 2 | 4.63 |
| Contig8512  (2001) | ND | GH9 | 32 | 0 | 4.05 |
| Contig21417  (2000) | ND | GH2 | 31 | 0 | 3.92 |
| Contig18616  (1988) | BD_contig02368  (1280) | GH94 | 33 | 0 | 4.2 |
| Contig20917  (1974) | ND | GH51 | 13 | 18 | 3.98 |
| Contig10363  (1969) | BD_contig02376  (1278) | GH9 | 7 | 22 | 3.74 |
| Contig22181  (1943) | BD_contig01721  (1476) | GH51-CBM_4_9 | 14 | 14 | 3.65 |
| Contig13811  (1929) | ND | GH3 | 20 | 1 | 2.75 |
| Contig17850  (1928) | BD_contig00989  (1854) | GH77 | 9 | 65 | 9.74 |
| Contig19988  (1901) | BD_contig03940  (1005) | GH31 | 18 | 10 | 3.73 |
| Contig20681  (1889) | BD_contig00941  (1889) | α-amylase-CBM48 | 32 | 10 | 5.63 |
| Contig18061  (1872) | BD_contig01641  (1503) | α-amylase-CBM48 | 20 | 11 | 4.2 |
| Contig18849  (1869) | BD_contig02321  (1291) | GH3 | 32 | 5 | 5.01 |
| Contig20055  (1830) | ND | GH43 | 27 | 0 | 3.73 |
| Contig13954  (1826) | ND | GH11-CBM6 | 35 | 1 | 4.99 |
| Contig18068  (1826) | BD_contig02512  (1249) | GH2 | 28 | 1 | 4.02 |
| Contig19642  (1811) | ND | GH43 | 16 | 12 | 3.92 |
| Contig17126  (1783) | BD_contig02665  (1216) | GH13 | 18 | 7 | 3.55 |
| Contig20296  (1739) | ND | GH94 | 17 | 6 | 3.35 |
| Contig13219  (1732) | BD_contig03133  (1132) | GH5-dockerin | 21 | 3 | 3.51 |
| Contig17365  (1712) | BD_contig02772  (1189) | GH57 | 4 | 48 | 7.71 |
| Contig18084  (1709) | BD_contig02523  (1249) | GH2 | 16 | 9 | 3.71 |
| Contig18878  (1708) | BD_contig02988  (1153) | GH9 | 15 | 14 | 4.3 |
| Contig23336  (1688) | BD_contig01023  (1655) | GH4 | 20 | 17 | 5.56 |
| Contig22510  (1672) | ND | α-amylase | 0 | 19 | 2.89 |
| Contig17388  (1653) | BD_contig01447  (1597) | GH43-CBM6 | 25 | 16 | 6.28 |
| Contig19516  (1625) | ND | GH2 | 11 | 8 | 2.96 |
| Contig19565  (1616) | BD_contig02906  (1165) | GH51 | 10 | 22 | 5.02 |
| Contig20783  (1613) | ND | GH31 | 7 | 15 | 3.46 |
| Contig13687  (1602) | ND | GH43-CBM6 | 9 | 18 | 4.28 |
| Contig23186  (1598) | ND | α-L-arabinofuranosidase | 15 | 1 | 2.53 |
| Contig16833  (1579) | ND | xylosidase | 13 | 5 | 2.89 |
| Contig13244  (1560) | ND | GH3 | 9 | 27 | 5.86 |
| Contig16737  (1556) | BD_contig02196  (1329) | GH31 | 38 | 3 | 6.67 |
| Contig10365  (1533) | ND | GH9 | 3 | 20 | 3.81 |
| Contig20327  (1522) | ND | GH4 | 22 | 6 | 4.66 |
| Contig21173  (1522) | ND | α-amylase | 1 | 15 | 2.67 |
| Contig18218  (1511) | ND | α-amylase | 1 | 12 | 2.18 |
| Contig17001  (1509) | BD_contig02566  (1234) | GH77-CBM20 | 0 | 21 | 3.53 |
| Contig18376  (1494) | BD_contig02049  (1367) | α-amylase | 10 | 36 | 7.81 |
| Contig22077  (1474) | ND | GH67 | 17 | 0 | 2.92 |
| Contig23802  (1462) | ND | GH98 | 2 | 10 | 2.08 |
| Contig19185  (1457) | ND | GH31 | 19 | 8 | 4.69 |
| Contig17698  (1450) | ND | GH67 | 10 | 3 | 2.27 |
| Contig21968  (1450) | ND | GH4 | 14 | 0 | 2.44 |
| Contig12527  (1430) | ND | GH3,GH3 | 4 | 19 | 4.08 |
| Contig18677  (1423) | ND | GH95 | 5 | 14 | 3.39 |
| Contig8998  (1421) | ND | GH5 | 13 | 0 | 2.31 |
| Contig13120  (1410) | ND | GH3 | 11 | 3 | 2.51 |
| Contig18571  (1407) | BD_contig02649  (1219) | GH18 | 18 | 0 | 3.24 |
| contig23023  (1401) | ND | GH67 | 7 | 18 | 4.53 |
| Contig19640  (1394) | BD_contig02728  (1195) | GH9 | 6 | 9 | 2.73 |
| Contig13239  (1381) | ND | GH43 | 10 | 4 | 2.57 |
| Contig21399  (1376) | ND | GH4 | 21 | 0 | 3.86 |
| Contig13862  (1361) | ND | GH3 | 5 | 9 | 2.61 |
| Contig18022  (1325) | ND | GH43-CBM6 | 18 | 0 | 3.44 |
| Contig19542  (1325) | ND | GH31 | 2 | 11 | 2.49 |
| Contig17827  (1323) | ND | GH67 | 17 | 0 | 3.25 |
| Contig22400  (1301) | ND | GH94 | 12 | 0 | 2.33 |
| Contig19184  (1300) | ND | GH94 | 18 | 0 | 3.5 |
| Contig20527  (1298) | ND | GH43-CBM_4_9 | 0 | 10 | 1.96 |
| Contig21799  (1297) | ND | GH57 | 19 | 0 | 3.71 |
| Contig12975  (1284) | ND | GH10-CBM9 | 11 | 0 | 2.17 |
| Contig19929  (1237) | BD_contig03727  (1035) | GH31 | 11 | 6 | 3.48 |
| Contig22495  (1234) | BD_contig00382  (1222) | GH109 | 0 | 24 | 4.94 |
| Contig8705  (1230) | ND | GH5 | 19 | 0 | 3.91 |
| Contig22836  (1215) | ND | GH31 | 18 | 0 | 3.75 |
| Contig8274  (1214) | ND | GH42 | 17 | 0 | 3.54 |
| Contig8316  (1200) | ND | GH9 | 15 | 0 | 3.16 |
| Contig19935  (1199) | ND | GH77 | 27 | 20 | 9.93 |
| Contig8411  (1191) | ND | GH9 | 17 | 0 | 3.61 |
| Contig22501  (1183) | ND | GH84 | 18 | 0 | 3.85 |
| Contig21256  (1172) | ND | xylosidase | 20 | 0 | 4.32 |
| Contig23111  (1167) | ND | GH43-CBM6 | 6 | 5 | 2.39 |
| Contig22341  (1162) | ND | α-amylase | 4 | 15 | 4.15 |
| Contig19897  (1159) | ND | GH8 | 12 | 1 | 2.84 |
| Contig23105  (1159) | ND | GH57 | 13 | 0 | 2.84 |
| Contig21644  (1154) | BD_contig02706  (1195) | α-amylase | 25 | 0 | 5.48 |
| Contig21425  (1142) | ND | α-L-arabinofuranosidase | 8 | 0 | 1.77 |
| Contig18850  (1133) | ND | GH115 | 14 | 4 | 4.02 |
| Contig20663  (1132) | ND | GH31 | 1 | 13 | 3.14 |
| Contig17664  (1125) | BD_contig03970  (1002) | α-amylase | 0 | 17 | 3.84 |
| Contig20069  (1125) | ND | GH2 | 14 | 0 | 3.15 |
| Contig18665  (1117) | ND | GH94-CBMX | 21 | 0 | 4.76 |
| Contig13562  (1115) | ND | GH3 | 3 | 10 | 2.96 |
| Contig8188  (1109) | ND | GH5 | 13 | 0 | 2.97 |
| Contig21187  (1108) | ND | GH51 | 13 | 0 | 2.97 |
| Contig10356  (1105) | ND | GH9 | 0 | 16 | 3.68 |
| Contig19839  (1105) | ND | GH57 | 0 | 18 | 4.14 |
| Contig17451  (1103) | ND | GH30-CBM6-dockerin | 19 | 0 | 4.36 |
| Contig21937  (1098) | ND | GH2 | 1 | 11 | 2.78 |
| Contig22184  (1095) | ND | GH43，GH95 | 4 | 8 | 2.78 |
| Contig17545  (1090) | ND | GH2 | 0 | 14 | 3.26 |
| Contig14199  (1086) | ND | GH3 | 10 | 0 | 2.33 |
| Contig21753  (1082) | ND | GH2 | 0 | 13 | 3.05 |
| Contig19533  (1079) | ND | GH43 | 7 | 9 | 3.76 |
| Contig19403  (1076) | ND | GH94 | 15 | 0 | 3.53 |
| Contig20746  (1076) | ND | GH97,GH92 | 22 | 0 | 5.17 |
| Contig21733  (1075) | ND | GH31,GH3 | 10 | 0 | 2.35 |
| Contig19560  (1058) | ND | GH94 | 10 | 0 | 2.39 |
| Contig9692  (1051) | ND | GH9-CBM3 | 11 | 0 | 2.65 |
| Contig19292  (1050) | ND | GH95 | 14 | 1 | 3.62 |
| Contig20272  (1046) | ND | GH2 | 13 | 1 | 3.39 |
| Contig22818  (1025) | ND | GH31 | 7 | 3 | 2.47 |
| Contig21649  (1024) | BD_contig02560  (1238) | GH57 | 6 | 15 | 5.2 |
| Contig23691  (1013) | ND | GH31 | 0 | 6 | 1.5 |
| Contig13669  (1010) | ND | GH3 | 2 | 5 | 1.76 |
| Contig20969  (1003) | ND | GH57 | 13 | 7 | 5.05 |
| Contig17189  (1000) | ND | GH13 | 4 | 10 | 3.55 |
| Contig21738  (999)^4^ | ND | GH65 | 0 | 10 | 2.54 |
| Contig22807  (995)^4^ | ND | GH3 | 5 | 9 | 3.57 |

^1^The GH-containing contigs assembled by the refinery assembly approach.

^2^The contigs ≥ 1Kb assembled by Newbler software corresponding to the GH-containing contigs; No contigs assembled by Newbler software ≥ 1Kb corresponding to the GH-containing contigs were shown as ND.

^3^Number of Z7 or Z8 metagenomic reads which used to assemble corresponding GH-containing contigs with the refinery assembly approach.

^4^These two GH-containing contigs were ≥ 1Kb during assembly process.
