# Supplementary material for: Insight into Dominant Cellulolytic Bacteria from Two Biogas Digesters and Their Glycoside Hydrolase Genes
Source: PLoS One. 2015 Jun 12;10(6):e0129921. doi: 10.1371/journal.pone.0129921 (PMC4466528; doi:10.1371/journal.pone.0129921)
Supplement: S13 Table — (DOCX) [file pone.0129921.s022.docx]

**S13 Table.** The 214 contigs assembled with Newbler software from the metagenomic reads of Z7 and Z8 assigned to BG-1 by PhyloPythiaS analysis.

| Contig ID | GH in the contigs | Length (bp) | Number of metagenomic reads^1^ | Depth |
| --- | --- | --- | --- | --- |
| BD_contig00160 | None | 4010 | 84 | 5.30 |
| BD_contig00206 | None | 3540 | 72 | 5.15 |
| BD_contig00264 | None | 3216 | 68 | 5.35 |
| BD_contig00297 | None | 3079 | 56 | 4.60 |
| BD_contig00324 | None | 2938 | 54 | 4.65 |
| BD_contig00369 | GH5 | 2795 | 56 | 5.07 |
| BD_contig00391 | None | 2748 | 40 | 3.68 |
| BD_contig00410 | None | 2696 | 51 | 4.79 |
| BD_contig00422 | None | 2648 | 42 | 4.01 |
| BD_contig00479 | None | 2540 | 48 | 4.78 |
| BD_contig00481 | None | 2527 | 52 | 5.21 |
| BD_contig00489 | None | 2498 | 54 | 5.47 |
| BD_contig00520 | None | 2413 | 50 | 5.24 |
| BD_contig00625 | None | 2221 | 34 | 3.87 |
| BD_contig00814 | None | 1990 | 37 | 4.70 |
| BD_contig00824 | None | 1977 | 33 | 4.22 |
| BD_contig00837 | None | 1974 | 42 | 5.38 |
| BD_contig00838 | None | 1973 | 49 | 6.28 |
| BD_contig00840 | None | 1969 | 35 | 4.50 |
| BD_contig00846 | None | 1963 | 36 | 4.64 |
| BD_contig00852 | None | 1958 | 36 | 4.65 |
| BD_contig00857 | None | 1955 | 47 | 6.08 |
| BD_contig00859 | None | 1955 | 34 | 4.40 |
| BD_contig00867 | None | 1949 | 41 | 5.32 |
| BD_contig00871 | None^2^ | 1946 | 36 | 4.68 |
| BD_contig00874 | None | 1944 | 39 | 5.08 |
| BD_contig00895 | None | 1926 | 43 | 5.65 |
| BD_contig00902 | None^2^ | 1921 | 49 | 6.45 |
| BD_contig00903 | None | 1914 | 58 | 7.67 |
| BD_contig00906 | None | 1918 | 36 | 4.75 |
| BD_contig00910 | None | 1911 | 34 | 4.50 |
| BD_contig00917 | None | 1902 | 31 | 4.12 |
| BD_contig00938 | None | 1892 | 28 | 3.74 |
| BD_contig00941 | None | 1889 | 41 | 5.49 |
| BD_contig00955 | None^2^ | 1880 | 33 | 4.44 |
| BD_contig00959 | None | 1875 | 47 | 6.34 |
| BD_contig00971 | None | 1868 | 36 | 4.88 |
| BD_contig00975 | None | 1863 | 33 | 4.48 |
| BD_contig00978 | None | 1863 | 37 | 5.02 |
| BD_contig00996 | None | 1840 | 27 | 3.71 |
| BD_contig01002 | None | 1847 | 47 | 6.44 |
| BD_contig01025 | None | 1828 | 39 | 5.40 |
| BD_contig01027 | None^2^ | 1827 | 34 | 4.71 |
| BD_contig01041 | None | 1819 | 34 | 4.73 |
| BD_contig01043 | None | 1821 | 45 | 6.25 |
| BD_contig01058 | None | 1813 | 33 | 4.61 |
| BD_contig01060 | None | 1812 | 39 | 5.45 |
| BD_contig01079 | None | 1804 | 34 | 4.77 |
| BD_contig01086 | None | 1801 | 41 | 5.76 |
| BD_contig01090 | None | 1798 | 36 | 5.07 |
| BD_contig01094 | None | 1795 | 37 | 5.22 |
| BD_contig01096 | None | 1793 | 40 | 5.64 |
| BD_contig01108 | None | 1790 | 31 | 4.38 |
| BD_contig01119 | None | 1784 | 28 | 3.97 |
| BD_contig01125 | None | 1779 | 43 | 6.12 |
| BD_contig01129 | None | 1778 | 43 | 6.12 |
| BD_contig01133 | None^2^ | 1773 | 34 | 4.85 |
| BD_contig01145 | None | 1761 | 96 | 13.79 |
| BD_contig01163 | None | 1750 | 55 | 7.95 |
| BD_contig01168 | None | 1746 | 58 | 8.40 |
| BD_contig01182 | None | 1742 | 26 | 3.78 |
| BD_contig01187 | None | 1735 | 30 | 4.37 |
| BD_contig01212 | None | 1725 | 40 | 5.87 |
| BD_contig01217 | None | 1705 | 57 | 8.46 |
| BD_contig01230 | None | 1710 | 43 | 6.36 |
| BD_contig01233 | None | 1712 | 28 | 4.14 |
| BD_contig01251 | None | 1705 | 27 | 4.01 |
| BD_contig01261 | None | 1699 | 30 | 4.47 |
| BD_contig01275 | None | 1686 | 35 | 5.25 |
| BD_contig01276 | None | 1688 | 33 | 4.95 |
| BD_contig01278 | GH10 | 1688 | 35 | 5.25 |
| BD_contig01280 | None | 1685 | 31 | 4.65 |
| BD_contig01295 | None | 1674 | 34 | 5.14 |
| BD_contig01303 | None | 1669 | 34 | 5.15 |
| BD_contig01315 | None | 1659 | 76 | 11.59 |
| BD_contig01326 | None | 1655 | 51 | 7.80 |
| BD_contig01328 | None | 1655 | 38 | 5.81 |
| BD_contig01333 | None | 1654 | 44 | 6.73 |
| BD_contig01340 | None | 1649 | 40 | 6.14 |
| BD_contig01345 | None | 1646 | 34 | 5.23 |
| BD_contig01355 | None | 1643 | 26 | 4.00 |
| BD_contig01380 | None | 1625 | 29 | 4.52 |
| BD_contig01393 | None | 1620 | 33 | 5.15 |
| BD_contig01396 | None | 1618 | 30 | 4.69 |
| BD_contig01406 | None | 1616 | 34 | 5.32 |
| BD_contig01408 | None | 1615 | 38 | 5.95 |
| BD_contig01442 | None^2^ | 1590 | 26 | 4.14 |
| BD_contig01450 | None | 1596 | 30 | 4.76 |
| BD_contig01456 | None | 1581 | 32 | 5.12 |
| BD_contig01477 | None | 1587 | 40 | 6.38 |
| BD_contig01484 | None | 1578 | 39 | 6.25 |
| BD_contig01486 | None | 1575 | 29 | 4.66 |
| BD_contig01498 | None | 1566 | 27 | 4.36 |
| BD_contig01512 | None | 1560 | 35 | 5.68 |
| BD_contig01537 | None | 1548 | 19 | 3.11 |
| BD_contig01560 | None | 1537 | 26 | 4.28 |
| BD_contig01570 | None | 1534 | 25 | 4.12 |
| BD_contig01581 | None | 1528 | 32 | 5.30 |
| BD_contig01586 | None | 1528 | 34 | 5.63 |
| BD_contig01601 | None | 1507 | 34 | 5.71 |
| BD_contig01617 | None | 1513 | 27 | 4.51 |
| BD_contig01618 | None | 1512 | 23 | 3.85 |
| BD_contig01638 | None | 1505 | 47 | 7.90 |
| BD_contig01641 | None | 1503 | 28 | 4.71 |
| BD_contig01684 | None | 1488 | 31 | 5.27 |
| BD_contig01703 | None | 1484 | 31 | 5.29 |
| BD_contig01708 | None | 1473 | 29 | 4.98 |
| BD_contig01752 | None | 1466 | 23 | 3.97 |
| BD_contig01773 | None | 1461 | 27 | 4.68 |
| BD_contig01795 | None | 1451 | 25 | 4.36 |
| BD_contig01811 | None | 1446 | 28 | 4.90 |
| BD_contig01813 | None | 1444 | 27 | 4.73 |
| BD_contig01816 | None | 1445 | 26 | 4.55 |
| BD_contig01819 | None | 1443 | 24 | 4.21 |
| BD_contig01824 | None | 1441 | 26 | 4.56 |
| BD_contig01839 | None | 1434 | 33 | 5.82 |
| BD_contig01861 | None | 1430 | 25 | 4.42 |
| BD_contig01865 | None | 1425 | 19 | 3.37 |
| BD_contig01894 | None | 1418 | 28 | 5.00 |
| BD_contig01902 | None | 1416 | 24 | 4.29 |
| BD_contig01913 | None | 1415 | 33 | 5.90 |
| BD_contig01915 | None | 1406 | 25 | 4.50 |
| BD_contig01919 | None | 1409 | 25 | 4.49 |
| BD_contig01960 | None | 1398 | 27 | 4.89 |
| BD_contig02010 | None | 1385 | 30 | 5.48 |
| BD_contig02016 | None | 1382 | 19 | 3.48 |
| BD_contig02034 | None | 1377 | 23 | 4.23 |
| BD_contig02048 | None | 1373 | 27 | 4.98 |
| BD_contig02057 | None | 1370 | 34 | 6.28 |
| BD_contig02064 | None | 1369 | 19 | 3.51 |
| BD_contig02075 | None | 1366 | 23 | 4.26 |
| BD_contig02084 | None | 1363 | 23 | 4.27 |
| BD_contig02167 | None | 1337 | 28 | 5.30 |
| BD_contig02180 | None | 1334 | 32 | 6.07 |
| BD_contig02192 | None | 1330 | 19 | 3.61 |
| BD_contig02196 | None | 1329 | 38 | 7.23 |
| BD_contig02267 | None | 1304 | 21 | 4.07 |
| BD_contig02280 | None | 1305 | 25 | 4.85 |
| BD_contig02311 | None | 1293 | 23 | 4.50 |
| BD_contig02313 | None | 1294 | 22 | 4.30 |
| BD_contig02321 | None | 1291 | 29 | 5.68 |
| BD_contig02328 | None | 1290 | 28 | 5.49 |
| BD_contig02329 | None | 1288 | 20 | 3.93 |
| BD_contig02359 | None | 1281 | 23 | 4.54 |
| BD_contig02363 | None | 1280 | 19 | 3.76 |
| BD_contig02364 | None | 1281 | 30 | 5.93 |
| BD_contig02374 | None | 1279 | 22 | 4.35 |
| BD_contig02394 | None | 1273 | 21 | 4.17 |
| BD_contig02398 | None | 1274 | 19 | 3.77 |
| BD_contig02435 | None | 1265 | 23 | 4.60 |
| BD_contig02472 | None | 1260 | 28 | 5.62 |
| BD_contig02485 | None | 1256 | 24 | 4.83 |
| BD_contig02511 | None | 1250 | 34 | 6.88 |
| BD_contig02516 | None | 1250 | 48 | 9.72 |
| BD_contig02524 | None | 1247 | 21 | 4.26 |
| BD_contig02529 | None | 1245 | 22 | 4.47 |
| BD_contig02534 | None | 1246 | 26 | 5.28 |
| BD_contig02553 | None | 1239 | 24 | 4.90 |
| BD_contig02577 | None | 1234 | 27 | 5.54 |
| BD_contig02598 | None | 1228 | 19 | 3.91 |
| BD_contig02609 | None | 1227 | 24 | 4.95 |
| BD_contig02622 | None | 1224 | 26 | 5.37 |
| BD_contig02635 | None | 1221 | 22 | 4.56 |
| BD_contig02643 | None | 1220 | 26 | 5.39 |
| BD_contig02651 | None | 1219 | 25 | 5.19 |
| BD_contig02654 | None | 1215 | 17 | 3.54 |
| BD_contig02655 | None | 1217 | 28 | 5.82 |
| BD_contig02664 | None | 1216 | 28 | 5.83 |
| BD_contig02678 | None | 1213 | 17 | 3.55 |
| BD_contig02703 | None | 1206 | 33 | 6.92 |
| BD_contig02747 | None | 1192 | 16 | 3.40 |
| BD_contig02756 | None | 1195 | 18 | 3.81 |
| BD_contig02805 | None | 1182 | 23 | 4.92 |
| BD_contig02816 | None | 1185 | 31 | 6.62 |
| BD_contig02853 | Putative GH | 1179 | 22 | 4.72 |
| BD_contig02867 | None^2^ | 1173 | 21 | 4.53 |
| BD_contig02926 | None | 1162 | 14 | 3.05 |
| BD_contig02952 | None | 1158 | 19 | 4.15 |
| BD_contig03069 | None | 1139 | 17 | 3.78 |
| BD_contig03074 | None | 1138 | 25 | 5.56 |
| BD_contig03119 | None | 1131 | 19 | 4.25 |
| BD_contig03135 | None | 1130 | 23 | 5.15 |
| BD_contig03138 | None | 1130 | 18 | 4.03 |
| BD_contig03139 | None | 1130 | 20 | 4.48 |
| BD_contig03154 | None | 1126 | 24 | 5.39 |
| BD_contig03208 | None | 1119 | 27 | 6.10 |
| BD_contig03215 | None^2^ | 1118 | 28 | 6.34 |
| BD_contig03253 | None | 1108 | 20 | 4.57 |
| BD_contig03318 | None | 1098 | 36 | 8.30 |
| BD_contig03389 | None | 1085 | 12 | 2.80 |
| BD_contig03415 | None | 1081 | 27 | 6.32 |
| BD_contig03417 | None | 1081 | 17 | 3.98 |
| BD_contig03439 | None | 1077 | 24 | 5.64 |
| BD_contig03468 | None | 1073 | 18 | 4.24 |
| BD_contig03487 | None | 1070 | 26 | 6.15 |
| BD_contig03505 | None | 1067 | 20 | 4.74 |
| BD_contig03529 | None | 1063 | 12 | 2.86 |
| BD_contig03549 | None | 1060 | 19 | 4.53 |
| BD_contig03595 | None | 1053 | 15 | 3.60 |
| BD_contig03630 | None | 1046 | 17 | 4.11 |
| BD_contig03648 | None | 1045 | 18 | 4.36 |
| BD_contig03657 | None | 1041 | 25 | 6.08 |
| BD_contig03660 | None | 1043 | 19 | 4.61 |
| BD_contig03711 | None | 1036 | 14 | 3.42 |
| BD_contig03726 | None | 1035 | 25 | 6.11 |
| BD_contig03727 | None | 1035 | 17 | 4.16 |
| BD_contig03780 | None | 1027 | 18 | 4.43 |
| BD_contig03815 | None | 1019 | 13 | 3.23 |
| BD_contig03819 | None | 1023 | 17 | 4.20 |
| BD_contig03841 | None | 1019 | 25 | 6.21 |
| BD_contig03885 | None | 1014 | 19 | 4.74 |
| BD_contig03909 | None | 1010 | 15 | 3.76 |
| BD_contig03948 | None | 1004 | 17 | 4.28 |
| BD_contig04008 | None | 1000 | 19 | 4.81 |

^1^Number of metagenomic reads used to assembled corresponding contigs

^2^Though there are no GH genes in the contigs, flagella related genes are present in the contigs.
