# Supplementary material for: Insight into Dominant Cellulolytic Bacteria from Two Biogas Digesters and Their Glycoside Hydrolase Genes
Source: PLoS One. 2015 Jun 12;10(6):e0129921. doi: 10.1371/journal.pone.0129921 (PMC4466528; doi:10.1371/journal.pone.0129921)
Supplement: S14 Table — (DOCX) [file pone.0129921.s023.docx]

**S14 Table.** Chemical analysis of slurry and methane yield of the two biogas digesters.

1. Chemical analysis data of slurry of the two biogas digesters.

| **Sample** | **Total nitrogen concentration in total solids^1^，%** | **Total carbon concentration in total solids^1^，%** | **C/N** | **NH_3_-N,** |
| --- | --- | --- | --- | --- |
|  |  |  |  | **g/L** |
| Slurry (Z7) | 2.569±0.032 | 34.239±0.206 | 13.33±0.08 | 1.61 |
| Slurry (Z8) | 2.574±0.001 | 35.269±0.374 | 13.70±0.14 | 1.67 |

1. Biogas production data of the two biogas digesters.

| **Sample** | **Methane production (L/week)^1^** |
| --- | --- |
|  |  |
| Z7 | 5.47±0.06 |
| Z8 | 2.11±0.16 |

^1^datas were measured for two times, including the fermentation cycle of Z7 and Z8 subjected to 454 sequencing and the following fermentation cycle.
